# Synthesis and characterization of fluorinated azadipyrromethene complexes as acceptors for organic photovoltaics

**DOI:** 10.3762/bjoc.12.182

**Published:** 2016-08-29

**Authors:** Forrest S Etheridge, Roshan J Fernando, Sandra Pejić, Matthias Zeller, Geneviève Sauvé

**Affiliations:** 1 Department of Chemistry, Case Western Reserve University, Cleveland, Ohio 44106, United States; 2 Department of Chemistry, Purdue University, West Lafayette, Indiana 47907, United States

**Keywords:** dye, fluorine, near-IR absorber, non-fullerene acceptor, zinc(II) complex

## Abstract

Homoleptic zinc(II) complexes of di(phenylacetylene)azadipyrromethene (e.g., Zn(WS3)_2_) are potential non-fullerene electron acceptors for organic photovoltaics. To tune their properties, fluorination of Zn(WS3)_2_ at various positions was investigated. Three fluorinated azadipyrromethene-based ligands were synthesized with fluorine at the *para*-position of the proximal and distal phenyl groups, and at the pyrrolic phenylacetylene moieties. Additionally, a CF_3_ moiety was added to the pyrrolic phenyl positions to study the effects of a stronger electron withdrawing unit at that position. The four ligands were chelated with zinc(II) and BF_2_^+^ and the optical and electrochemical properties were studied. Fluorination had little effect on the optical properties of both the zinc(II) and BF_2_^+^ complexes, with λ_max_ in solution around 755 nm and 785 nm, and high molar absorptivities of 100 × 10^3^ M^−1^cm^−1^ and 50 × 10^3^ M^−1^cm^−1^, respectively. Fluorination of Zn(WS3)_2_ raised the oxidation potentials by 0.04 V to 0.10 V, and the reduction potentials by 0.01 V to 0.10 V, depending on the position and type of substitution. The largest change was observed for fluorine substitution at the proximal phenyl groups and CF_3_ substitution at the pyrrolic phenylacetylene moieties. The later complexes are expected to be stronger electron acceptors than Zn(WS3)_2_, and may enable charge transfer from other conjugated polymer donors that have lower energy levels than poly(3-hexylthiophene) (P3HT).

## Introduction

**Figure 1:**
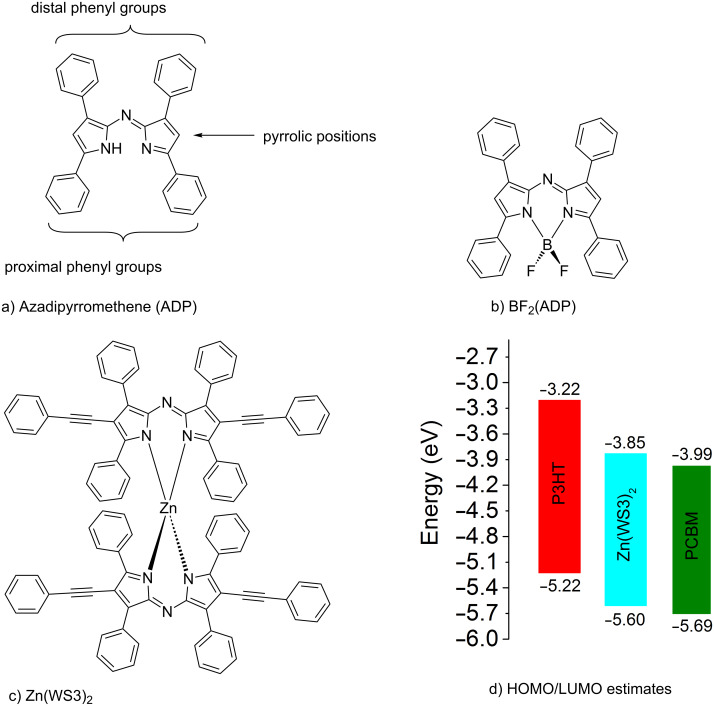
a) Azadipyrromethene ligand labeling positioning; b and c) chelates; d) estimated HOMO/LUMO energy levels [9].

Azadipyrromethenes (ADPs) (Figure 1a) are a class of monoanionic bidentate ligands with strong absorption in the visible and near IR range. Their absorbance and emission properties can be readily tuned through structural modifications and chelation [1–4]. BF_2_^+^-chelated ADP derivatives (Figure 1b) in particular have drawn interest for photodynamic therapy, bio-imaging and light harvesting applications [5–8].

We have shown that derivatives of Zn(ADP)_2_ are promising electron acceptors for organic photovoltaics (OPVs) [9–10]. A series of zinc(II) derivatives with various pyrrolic substitutions showed power conversion efficiencies (PCEs) ranging from 2.2–4.1% when blended with poly(3-hexylthiophene) (P3HT), and the highest PCE was obtained with Zn(WS3)_2_, shown in Figure 1c [9–10]. In comparison, free ligands and BF_2_^+^ chelates showed negligible power conversion efficiencies, and Zn(ADP)_2_ gave a maximum efficiency of 1.4% [11]. Free ligands and BF_2_^+^ chelates tend to self-aggregate too much, leading to large scale phase separation with P3HT and low PCEs. Chelation with zinc(II) lowers the tendency to self-aggregate, but Zn(ADP)_2_ still shows some large aggregates in blend films [10]. The addition of pyrrolic phenylacetylene substituents further helped to break the aggregation and to create a favorable nanoscale phase separation in blend films, leading to higher PCEs [9–10]. DFT calculations show that Zn(WS3)_2_ is a very large and non-planar molecule with low electron transfer reorganization energy, further supporting its potential to replace fullerenes in OPVs [12]. However, the HOMO and LUMO energy levels of Zn(WS3)_2_ in solution are higher than that of the most popular electron acceptor phenyl-C_61_-butyric acid methyl ester (PCBM, Figure 1d). Decreasing the energy levels of the parent Zn(WS3)_2_ would enable this new class of acceptors to be paired with other polymer donors that have lower HOMO and LUMO energy levels than P3HT.

One substituent used to modify the properties of molecules without drastically altering the structure is fluorine, which is a unique element due to its small size and high electronegativity, with many applications in pharmaceuticals and materials [13–15]. Its use as a hydrogen replacement has garnered wide use in industrial applications for high thermal stability and surface effects, most notably with polytetrafluoroethylene (PTFE) [16]. The small size and strong electron-withdrawing properties of fluorine make it ideally suited to tune the molecular orbital (MO) energy levels of polymer donors in OPVs without major influence on the structure [17–22]. In several cases, the addition of fluorine decreases the energy of the highest occupied molecular orbitals (HOMO), thereby enhancing the open-circuit voltages (*V*_oc_) and PCEs [17,23–26]. A 2014 investigation by Luscombe and co-workers showed that a fluorine substitution lowered the charge transfer exciton (CTE) binding energy, in turn creating more free carriers and higher PCEs [19]. Additionally, enhancements to the short-circuit current density (*J*_sc_), *V*_oc_, and fill-factor (FF) in OPVs have been attributed to the addition of fluorine substituents [17–18,20–21,27–29].

While fluorinated polymer donors are well known, fluorinated n-type materials are less common [13,30–31]. Nevertheless, fluorinated n-type materials have been shown to exhibit promising characteristics in devices. For instance, the addition of fluorinated groups to naphthalene diimide (NDI) and perylene diimide (PDI) derivatives increased the reduction potentials (more positive), allowed for air-stable fabrication of organic field effect transistors (OFETs), and showed promise in solar cells [13,32]. Some homoleptic metal complexes, such as Ir(III) phenylpyridine-based complexes, are favored for their use in light-emitting devices and the fluorinated derivatives allow access to triplet state blue light-emitters [13].

**Figure 2:**
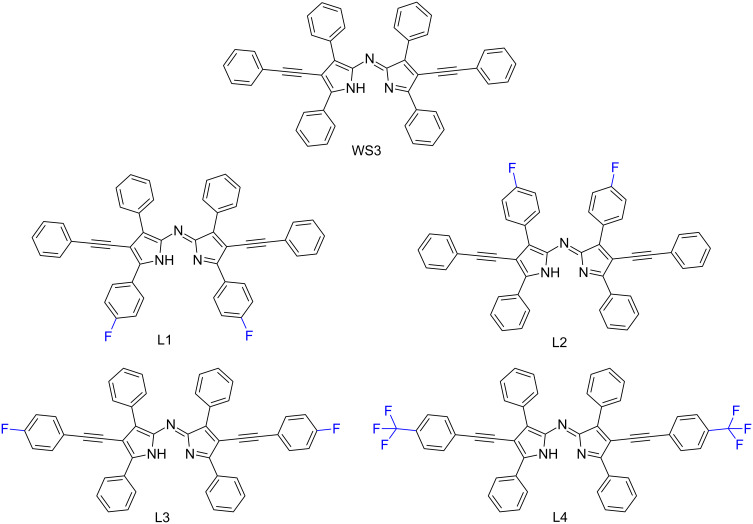
Chemical structures of the fluorinated ADP derivatives of WS3 explored in the study.

This work further investigates the effects of fluorination in n-type materials for OPVs. A series of selectively fluorinated ADP derivatives based on WS3 were synthesized (Figure 2). To understand the effect of the fluorination position, a single fluorine atom was added at three places: at the proximal phenyl position (L1), at the distal phenyl group (L2), and at the pyrrolic phenylacetylene moiety (L3). At the latter position, the degree of fluorination was further explored with the addition of CF_3_ (L4). These four fluorinated derivatives were then chelated with both BF_2_^+^ and zinc(II) (Scheme 2, see below), and their optical and electrochemical properties were studied. The BF_2_^+^ chelates were included in this study because they may be useful near-IR absorbers for other light-harvesting applications. The fluorine substitutions had little effect on the optical properties of the BF_2_^+^ and zinc(II) chelates, and only had a small effect on the electrochemical properties, with the largest increase in oxidation and reduction potentials of 0.1 V observed for L1 and L4 chelates compared to WS3 chelates. Zinc(II) chelates of L1 and L4 are expected to be stronger electron acceptors than Zn(WS3)_2_, and may enable charge transfer from other conjugated polymer donor that have lower energy levels than P3HT.

## Results and Discussion

### Synthesis

**Scheme 1:**
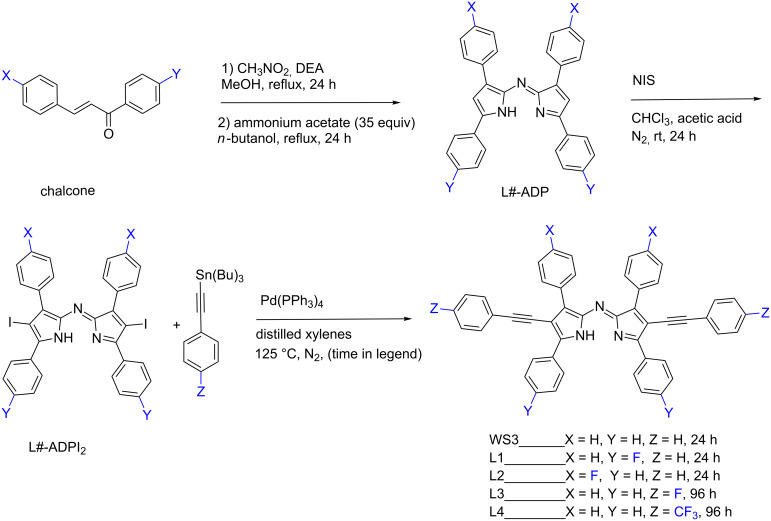
Generic synthetic scheme for fluorinated free ligands, where L# corresponds to the desired ligand number. For instance, L3-ADP and L3-ADPI_2_ would lead to the synthesis of the free ligand L3.

The synthesis of ADP was carried out according to literature procedures [9,33]. The ADP-analogs with fluorine at the proximal or distal phenyl positions (L1-ADP and L2-ADP) were synthesized in a similar fashion with the respective fluorinated chalcones (Scheme 1). In this case, the nitro intermediates of the fluorinated chalcones could not be isolated as a solid powder, so the synthesis was carried forward assuming complete conversion from the first reaction.

To install phenylacetylene groups, iodination of the ADP derivatives was done according to literature procedures and purified by washing with chloroform in good yield [5,33–34]. Stille cross-coupling with the appropriate tributyltin-phenylacetylene analogs afforded the WS3 derivatives in good yield (Scheme 1). We chose to utilize Stille coupling instead of Sonogashira coupling because we had previously found that this method gives higher yields for installing phenylethynyl pyrrolic substituents [9]. The fluorinated tributyltinphenylacetylene analogs for the synthesis of L3 and L4 were synthesized according to literature procedures and used without purification [35–36]. The Stille cross coupling reactions for the synthesis of L3 and L4 were monitored by MALDI–TOF–MS and were found to not be complete after increasing the reaction time to 48 h, so the reaction time was increased to 96 h with the addition of more catalyst and tributyltin reactant after 48 h. These modifications were deemed necessary to push the reaction towards completion and aid in purification of the free ligand. The free ligands were isolated from the crude mixture by rotary evaporation and purified by trituration with cold methanol and the remaining solid was washed with cold ether. Due to the lowered solubility of the iodinated ADP derivatives and the free ligands in organic solvents, the identity of these compounds was confirmed only by MADLI–TOF–MS. These modifications allowed for the synthesis of all fluorinated WS3 derivatives in good yield with sufficient purity for chelation.

**Scheme 2:**
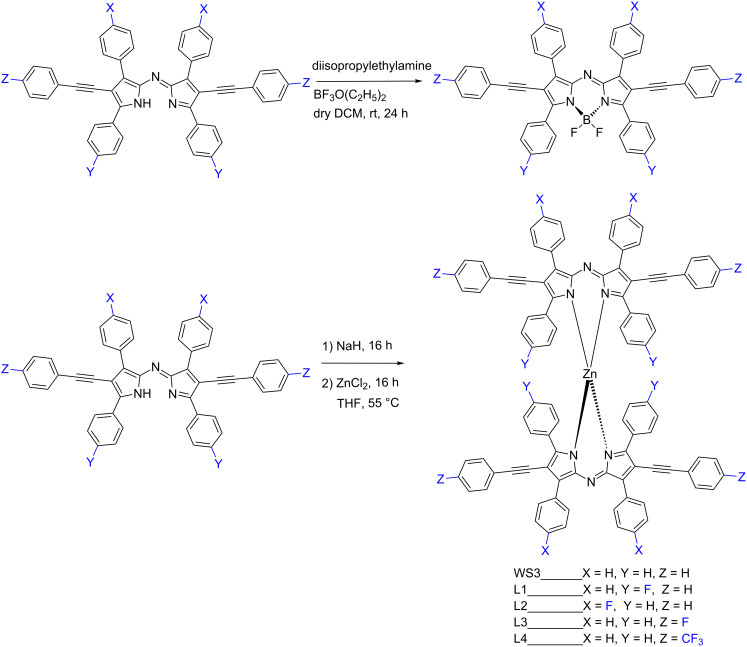
Generic chelation scheme yielding WS3-based BF_2_^+^ and zinc(II) complexes.

The BF_2_^+^ chelation was carried out according to the literature procedures in moderate yields (Scheme 2) [2,5]. For the zinc(II) chelation, the reaction was changed from a reaction using Zn(OAc)_2_ to a 2-step, one pot reaction with sodium hydride in tetrahydrofuran, followed by the addition of zinc(II) chloride. Zinc(II) and BF_2_^+^ chelates were purified by silica gel column chromatography to isolate the chelates as blue solids and the identity and purity was confirmed by NMR spectroscopy, MALDI–TOF–MS and elemental analysis. In the case of L2, the pure BF_2_^+^ chelate could not be isolated by column chromatography, and will therefore be omitted from further analysis.

**Figure 3:**
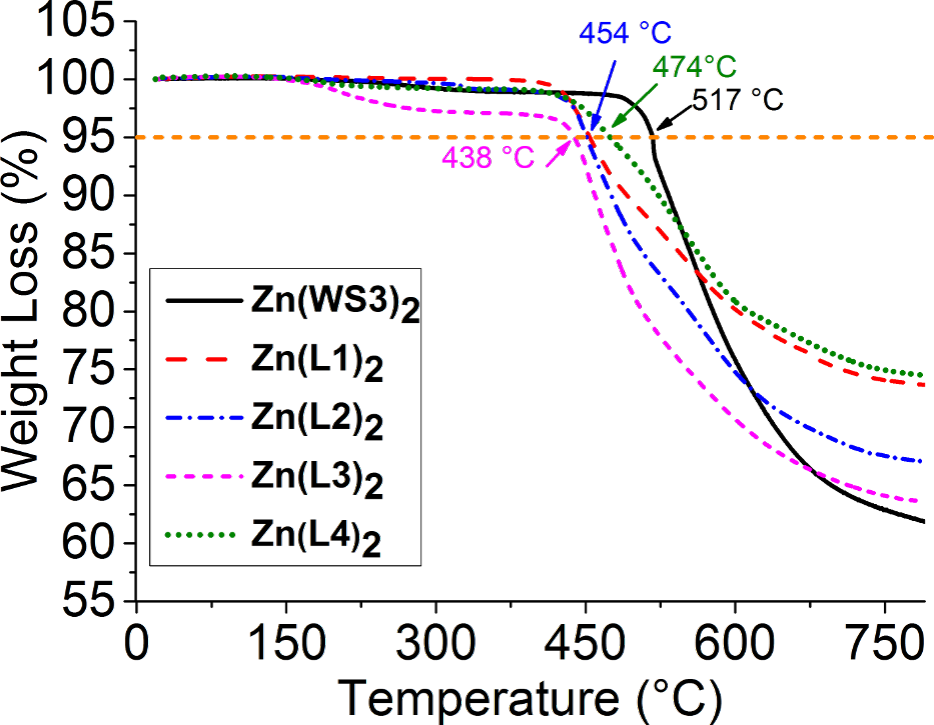
TGA spectra for the zinc(II) complexes.

The thermal stability of the zinc(II) complexes was examined by thermal gravity analysis and the results are shown in Figure 3. The fluorinated complexes had weight loss profiles similar to each other with a 5% loss between 438 °C and 474 °C, all lower than that of the unfluorinated Zn(WS3)_2_ at 517 °C.

### Optical properties

**Figure 4:**
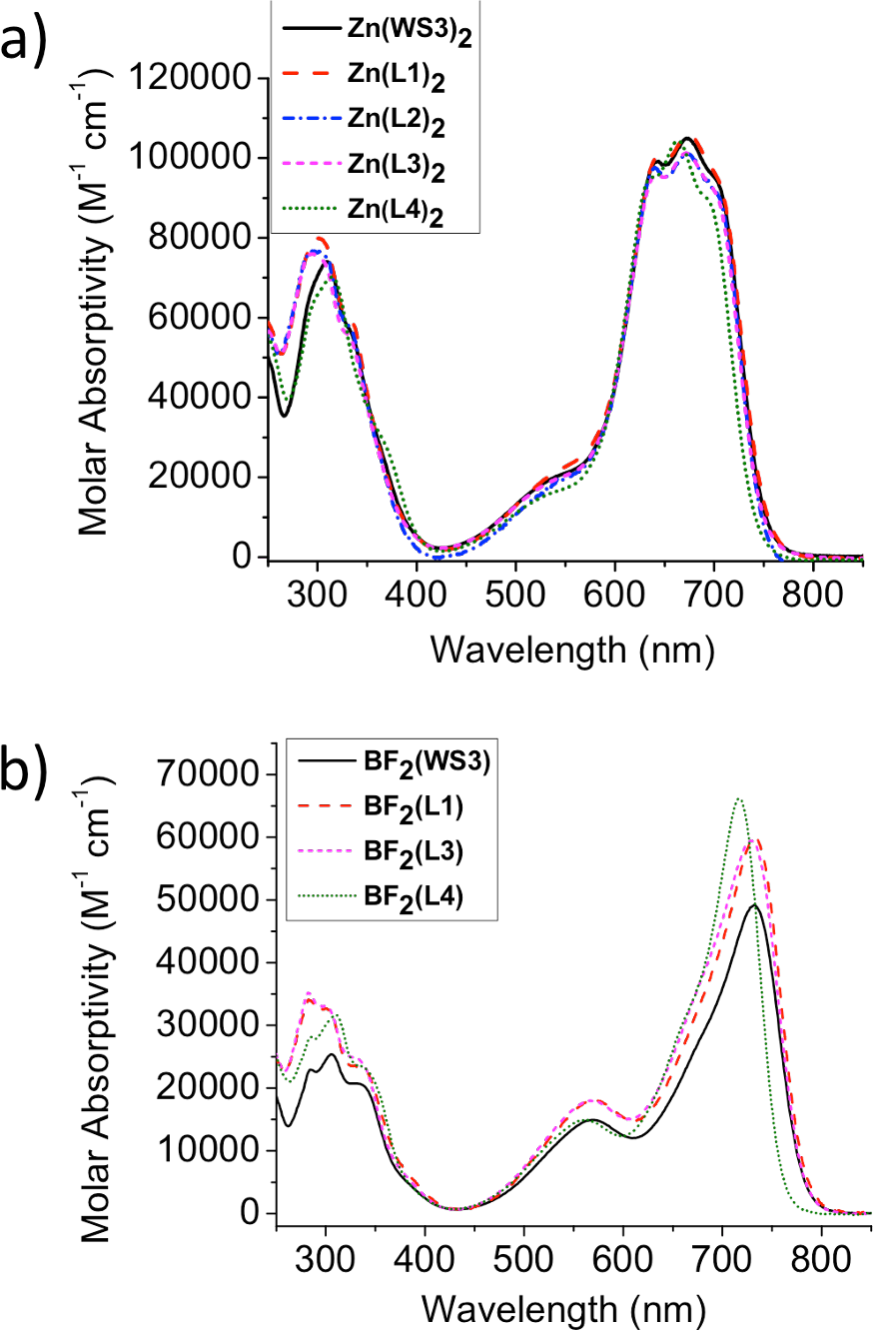
Molar absorptivities in chloroform solutions of a) zinc(II) chelates b) BF_2_^+^ chelates.

Optical studies for the zinc(II) and BF_2_^+^ complexes were performed in chloroform solutions and with spun-cast films on microscope slides. Like the solid powders, all of the solutions and films were dark blue. Solution and film optical properties for zinc(II) and BF_2_^+^ chelates are summarized in Table 1. The molar absorptivity spectra in chloroform solutions for zinc(II) and BF_2_^+^ chelates are reported in Figure 4a and Figure 4b, respectively.

**Table 1:** Summary of optical properties of zinc(II) and BF_2_^+^ chelates in solution and film.

	Solution		Film	
	λ_max_ (nm) (ε,× 10^3^ M^−1^cm^−1^)	λ_onset_ (nm)	λ_max_ (nm)	λ_onset_ (nm)

Zn(WS3)_2_	310(74), 664(99), 674(105)	757	696	791
BF_2_(WS3)	732(49)	782	759	835
Zn(L1)_2_	302(80), 643(100), 674(106)	759	695	785
BF_2_(L1)	732(60)	783	755	829
Zn(L2)_2_	302(77), 640(98), 672(101)	757	697	780
BF_2_(L2)	–	–	–	–
Zn(L3)_2_	294(76), 642(96), 672(101)	755	692	778
BF_2_(L3)	729(59)	783	770	868
Zn(L4)_2_	314(70), 663(104)	746	676	769
BF_2_(L4)	717(66)	763	669	800

The absorption spectra of the zinc(II) chelates are all similar. In most cases, the λ_max_ and λ_onset_ values remain consistent with Zn(WS3)_2_ around 670 nm and 755 nm, respectively. An exception is Zn(L4)_2_, with λ_max_ and λ_onset_ blue-shifted by 10 nm compared to Zn(WS3)_2_. This hypsochromic shift is consistent with other cases where a highly polarized CF_3_ group is added *para* to the conjugated structure [13,37]. Regardless of fluorination, the extinction coefficients are all near 100 × 10^3^ M^−1^cm^−1^, showing the strong absorption properties of the WS3-core.

Solutions of BF_2_^+^ chelates show a consistent trend compared with the solutions of zinc(II) chelates. The λ_max_ and λ_onset_ values of BF_2_(WS3), BF_2_(L1), and BF_2_(L3) are ≈730 nm and ≈780 nm, respectively. Consistent with that of the zinc(II) chelate solutions, λ_max_ of BF_2_(L4) is 15 nm blue shifted compared to BF_2_(WS3), showing a slightly greater effect from CF_3_ in the BF_2_^+^ chelate. Molar absorptivities of the compounds vary from 49 × 10^3^ M^−1^cm^−1^ for BF_2_(WS3) to 66 × 10^3^ M^−1^cm^−1^ for BF_2_(L4).

**Figure 5:**
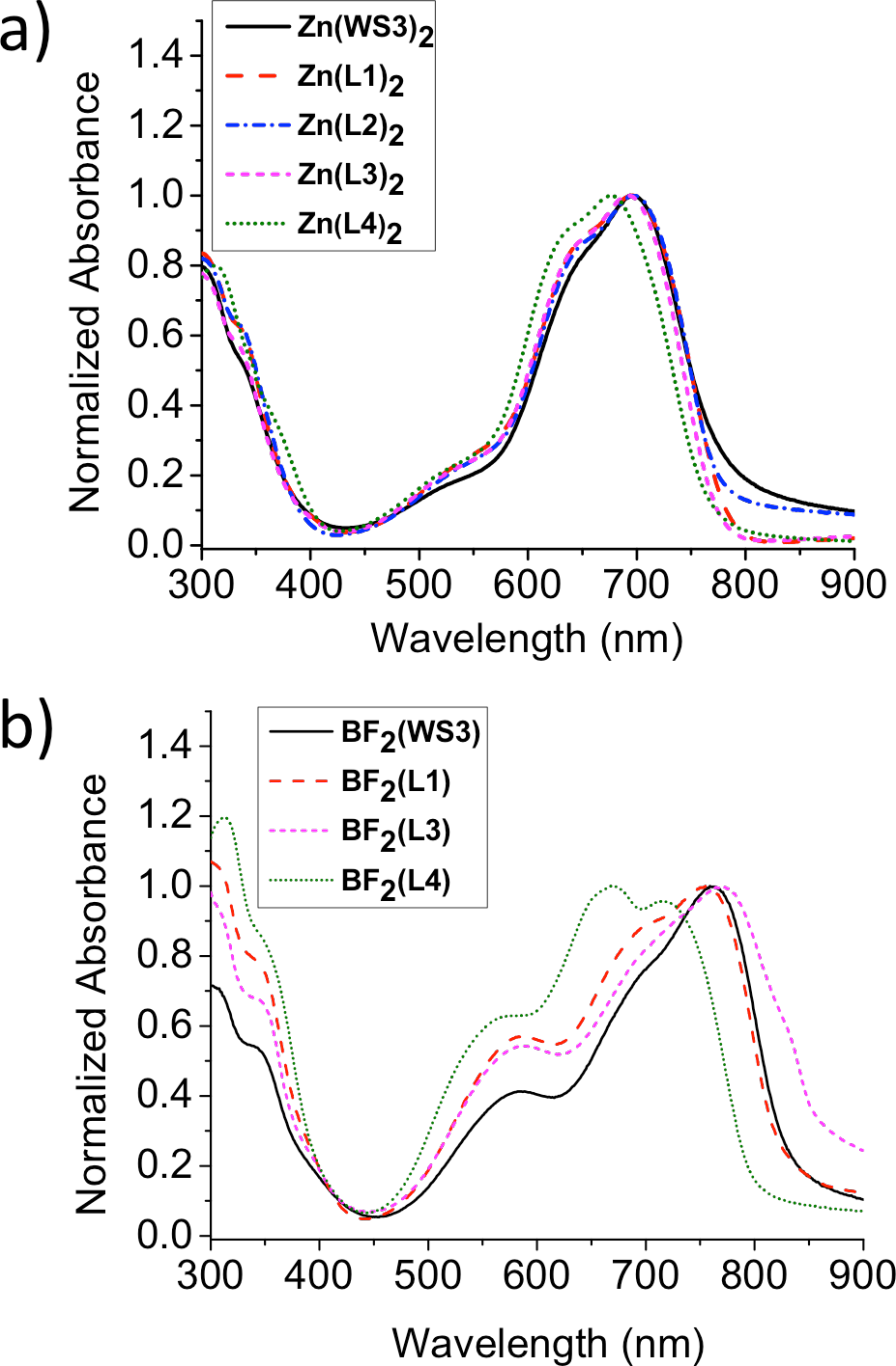
Normalized absorbance from spun-coat chloroform solution on microscope glass of a) zinc(II) chelates b) BF_2_^+^ chelates.

Films of the zinc(II) and BF_2_^+^ chelates were made in order to better understand the optical properties of the materials in devices, and the properties are summarized in Table 1. Normalized absorption spectra of zinc(II) and BF_2_^+^ chelate films are reported in Figure 5a and Figure 5b, respectively. Following the same trend as the zinc(II) chelate solutions, the zinc(II) chelate films exhibited consistent λ_max_ values of ≈695 nm, excluding Zn(L4)_2_ at 676 nm. The λ_onset_ values for films of Zn(L1)_2_, Zn(L2)_2_, Zn(L3)_2,_ and Zn(L4)_2_ are 785, 780, 778, and 769 nm, respectively, all blue shifted compared to λ_onset_ of Zn(WS3)_2_ at 791 nm. The shapes of all the curves remain consistent with a broad absorption from 500 to 800 nm, good for OPV applications.

All BF_2_^+^ chelate films exhibit broadening compared to solutions, with an absorbance ranging from 450 to 900 nm. The λ_max_ values for BF_2_(WS3) and BF_2_(L1) are similar at 759 and 755 nm, respectively. The onset values for the two films are also similar at 835 and 829 nm, respectively. Films of BF_2_(L3) and BF_2_(L4) show marked differences from two different fluorine modifications at the same position. In BF_2_(L3), the λ_max_ and λ_onset_ are recorded at 770 and 868 nm, respectively. Compared to BF_2_(WS3), there is a small 11 nm bathochromic shift of λ_max_. For BF_2_(L4), λ_max_ and λ_onse_ are observed at 669 and 800 nm, respectively. Compared to λ_max_ of BF_2_(WS3), BF_2_(L4) exhibits a large hypsochromic shift of 33 nm.

### Electrochemistry

**Figure 6:**
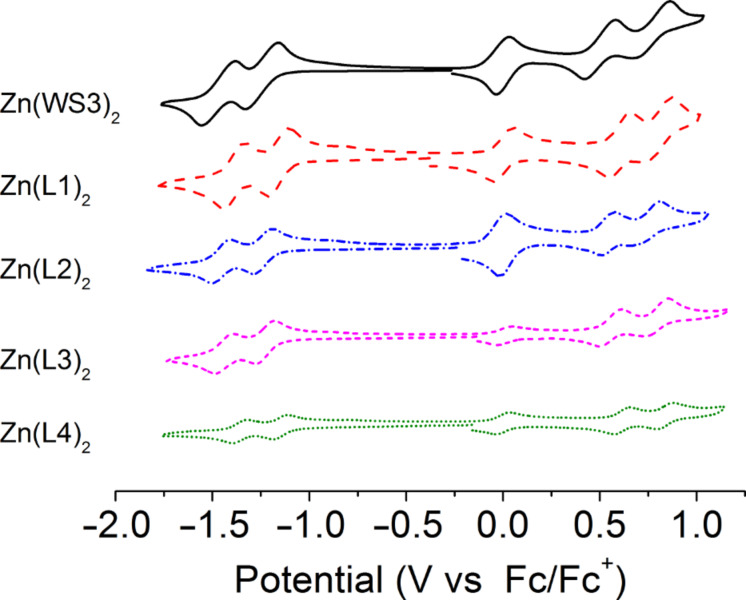
Cyclic voltamograms of zinc(II) chelates in 0.1 M TBAPF_6_ dichloromethane solution with Fc/Fc^+^ as an internal standard (*E*_1/2_ at 0.0 V).

**Figure 7:**
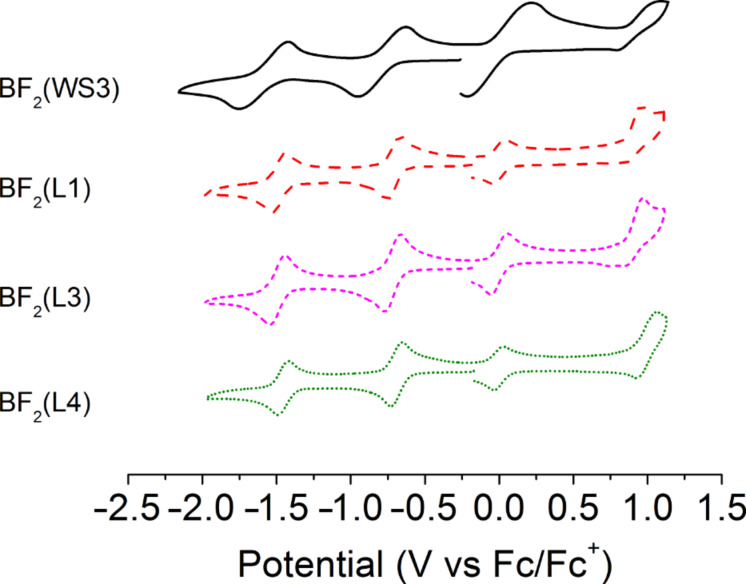
Cyclic voltamograms of BF_2_^+^ chelates in 0.1 M TBAPF_6_ dichloromethane solution with Fc/Fc^+^ as an internal standard(*E*_1/2_ at 0.0 V).

Cyclic voltammetry of the zinc(II) and BF_2_^+^ chelates was studied in dichloromethane solutions using ferrocene/ferricinium (Fc/Fc^+^) as an internal reference. The electrochemical properties of the complexes are summarized in Table 2 with the voltammograms of the zinc(II) and BF_2_^+^ chelates shown in Figure 6 and Figure 7, respectively. For all zinc(II) complexes, cyclic voltammograms reveal two reversible oxidations, while an irreversible oxidation occurs for all BF_2_^+^ chelates. The first oxidation potentials (*E*_1/2 ox._) of the fluorinated zinc(II) chelates were higher than that of Zn(WS3)_2_ (0.50 V) by at least 0.04 V, with the highest value being 0.61 V. The increased *E*_1/2 ox._ values are consistent with the increased oxidative stability afforded by the addition of fluorine [13]. The second oxidation potential for all zinc(II) chelates, 0.77–0.79 V, showed little change except in two cases: Zn(L4)_2_ and Zn(L2)_2_ had second oxidation potentials of 0.84 V and 0.73 V, respectively. The differences between the first and second oxidation potential was 0.27 V for Zn(WS3)_2_, while it was 0.18 V and 0.19 V for Zn(L1)_2_ and Zn(L2)_2_, respectively. For both Zn(L3)_2_ and Zn(L4)_2_ the difference between oxidation potentials was 0.23 V, a slight decrease from Zn(WS3)_2_ at 0.27 V. All of the fluorinated zinc(II) complexes exhibit a rise of the first oxidation potential as well as a decrease between the first and second oxidation potentials, compared to Zn(WS3)_2_.

**Table 2:** Electrochemical properties of zinc(II) and BF_2_^+^ chelates in dichloromethane. All values reported are in V vs Fc/Fc^+^.

	*E*_1/2 ox._	*E*_(p,a)_	*E*_1/2 red._	*E*_(p,c)_

Zn(WS3)_2_	0.50, 0.77	0.58, 0.86	−1.25, −1.47	−1.33, −1.55
BF_2_(WS3)	−	1.08	−0.79, −1.59	−0.95, −1.75
Zn(L1)_2_	0.60, 0.78	0.66, 0.87	−1.16, −1.39	−1.11, −1.33
BF_2_(L1)	−	0.96	−0.71, −1.48	−0.67, −1.44
Zn(L2)_2_	0.54, 0.73	0.58, 0.81	−1.24, −1.45	−1.19, −1.41
BF_2_(L2)	–	–	–	–
Zn(L3)_2_	0.56, 0.79	0.61, 0.86	−1.23, −1.44	−1.18, −1.39
BF_2_(L3)	−	0.97	−0.72, −1.49	−0.66, −1.44
Zn(L4)_2_	0.61, 0.84	0.66, 0.88	−1.15, −1.36	−1.11, −1.32
BF_2_(L4)	−	1.06	−0.69, −1.46	−0.65, −1.42

Cyclic voltammograms of both the zinc(II) and BF_2_^+^ complexes showed two reversible reduction potentials. The reduction potentials (*E*_1/2 red._) of Zn(L2)_2_ and Zn(L3)_2_ were similar to that of Zn(WS3)_2_, suggesting that the addition of one fluorine atom at the pyrrolic phenylacetylene or distal phenyl position does not stabilize the anion. On the other hand, there is significant increase of the reduction potentials going from −1.25 V to −1.16 V and −1.15 V for Zn(WS3)_2_, Zn(L1)_2_ and Zn(L4)_2_, respectively. This suggests that fluorine stabilizes the anion when at the proximal position or when a CF_3_ group is installed at the pyrrolic phenylacetylene moiety. The difference between the first oxidation and first reduction potentials of all the zinc(II) complexes are similar, 1.75 V to 1.79 V. This means that in the cases of Zn(L1)_2_ and Zn(L4)_2_, fluorine has a similar stabilizing effect on both the cation and anion.

**Figure 8:**
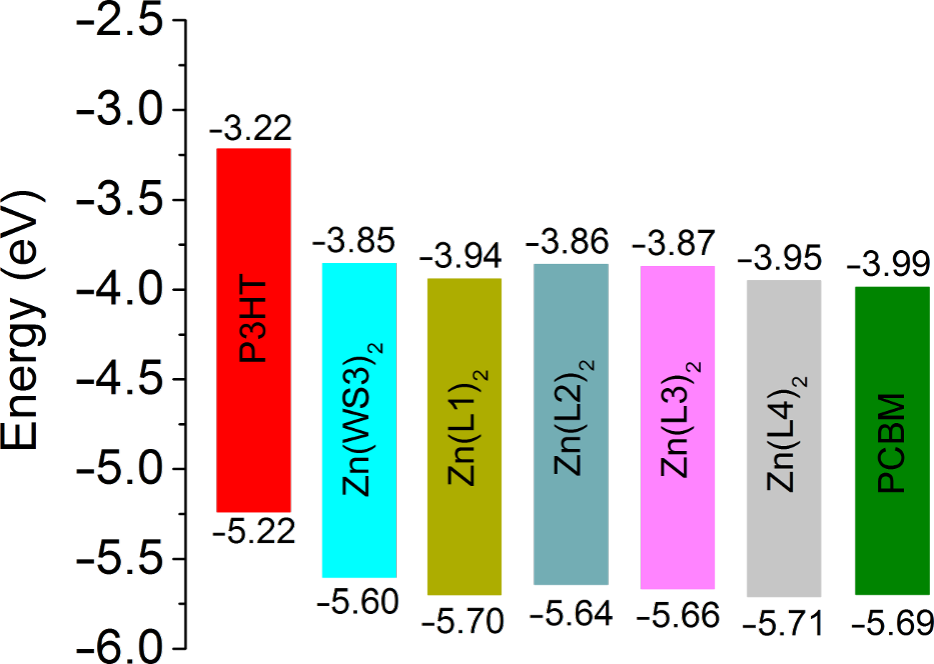
Estimated HOMO and LUMO energy levels obtained by cyclic voltammetry from the *E*_1/2_ values in dichloromethane solution, using the value of −5.1 eV for Fc/Fc^+^. The included HOMO and LUMO levels of P3HT films were estimated in our laboratory from the oxidation onset and the optical gap.

The differences between the first and second reduction potentials of each compound were similar, indicating that fluorine influences both reductions equally. For the fluorinated BF_2_^+^ complexes, the first and second reduction potentials were slightly more positive than those of BF_2_(WS3). The *E*_1/2 red._ values ranged from −0.79 V for BF_2_(WS3) to −0.69 V for BF_2_(L4). All the first reduction potentials of the fluorinated BF_2_^+^ complexes were −0.70 V with the difference between first and second *E*_1/2_ values being 0.77 V. Collectively, the fluorine-modified WS3 chelates showed higher oxidation potentials than those of unmodified chelates. The interpretation of electrochemical data shows that the addition of fluorine had the greatest effect on L1 and L4 chelates, while having minimal effects on the L2 and L3 chelates. The estimated HOMO and LUMO energy levels obtained from cyclic voltammetry are shown in Figure 8. The HOMO and LUMO of Zn(L1)_2_ and Zn(L4)_2_ approach those of PCBM.

### Crystallography

**Figure 9:**
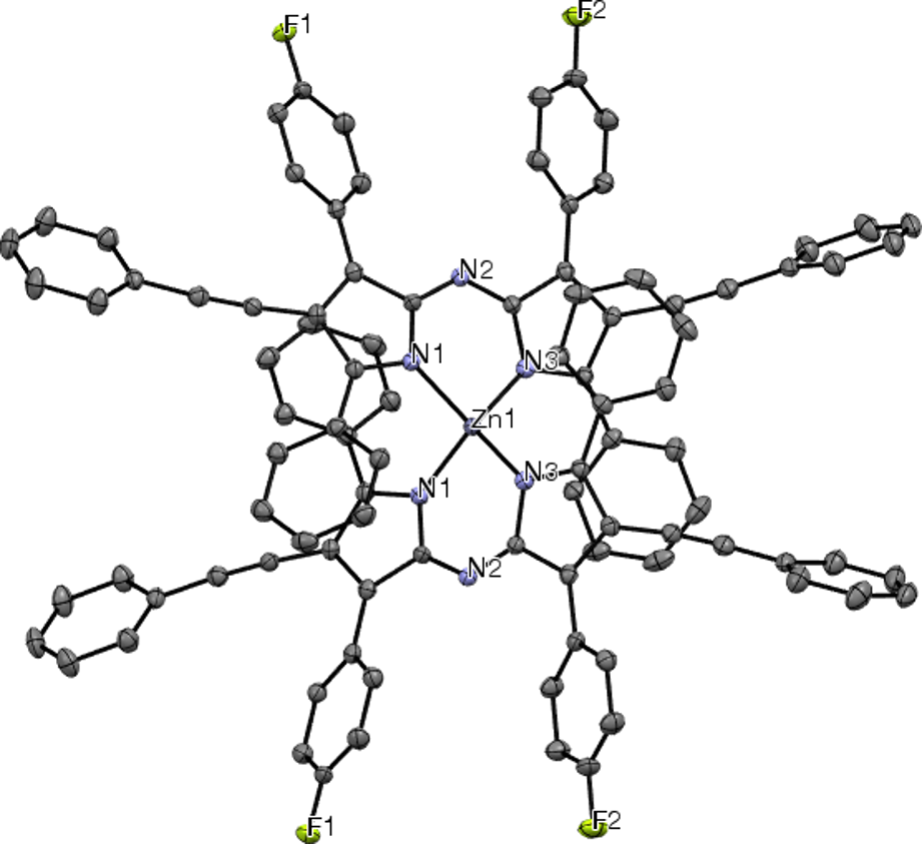
ORTEP drawing of Zn(L2)_2_ with ellipsoids drawn at the 50% probability level and a partial labeling scheme. The hydrogen atoms, and dichloromethane solvate were omitted for clarity.

**Figure 10:**
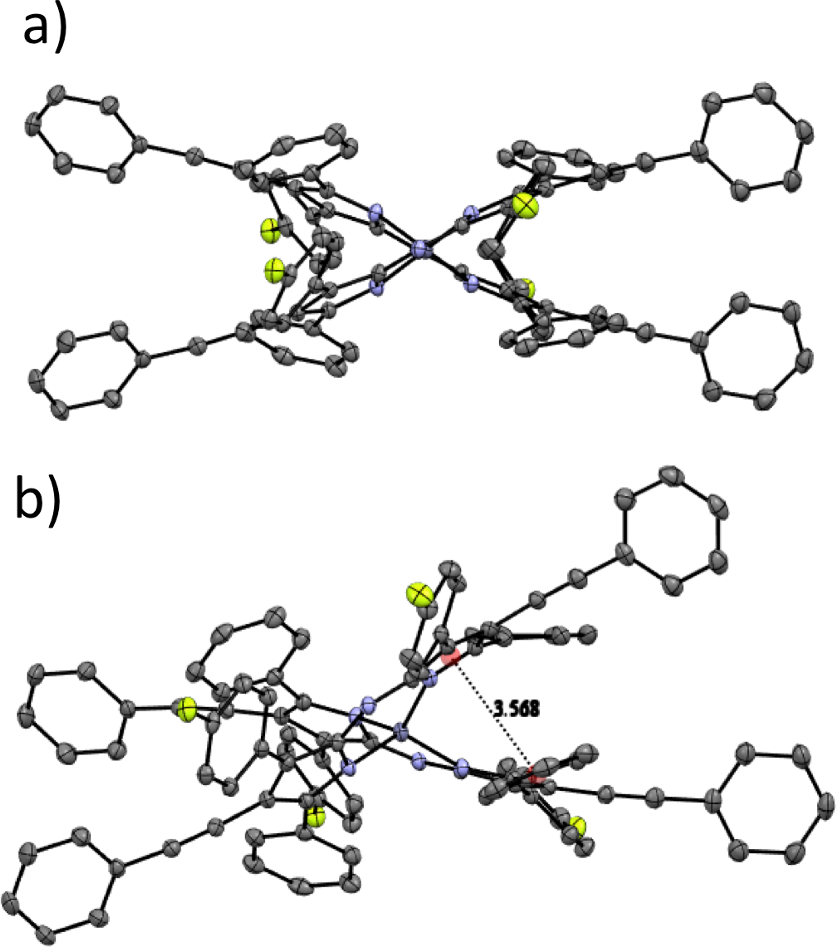
ORTEP drawing of Zn(L2)_2_ with ellipsoids drawn at the 50% probability level and a partial labeling scheme. The hydrogen atoms, and dichloromethane solvate were omitted for clarity. a) Shows the distorted tetrahedral shape; b) Shows the π-stacking between the proximal phenyl group of one ligand and a pyrrole ring of the opposite ligand.

Single crystals of the zinc(II) chelates were grown in order to better understand the structure of the materials. Only Zn(L2)_2_ produced crystals suitable for analysis. Figure 9 shows the ORTEP drawing of Zn(L2)_2_ with 50% ellipsoids and a partial labeling scheme for clarity. The crystal structure confirms the identity of the complex and gives an idea of the interactions in the complex. Like Zn(ADP)_2_, the structure is distorted tetrahedral with favorable π–π stacking distances between the proximal phenyl and pyrrole rings of the two separate ligands (Figure 10a and 10b). The distance between centroids is 3.56 Å for Zn(L2)_2_, compared to 3.63 Å for Zn(ADP)_2_ [38]. The shorter distance found for Zn(L2)_2_ suggests a stronger interaction between the proximal phenyl and pyrrole rings than in Zn(ADP)_2_. Unfortunately, it cannot be determined whether the addition of fluorine or phenylacetylene contributed to the shorter π–π stacking distances without a crystal structure for Zn(WS3)_2_. Intermolecular favorable π–π stacking distances are observed between the pyrrolic phenylacetylene arms of two chelates seen on the outside of the unit cell, as well as between the distal phenyl rings of two chelates. Due to the crowded packing and difficulty in obtaining a clear image to convey these observations, the authors invite the reader to observe the intermolecular packing on their own using the cif file provided as Supporting Information File 1.

### Preliminary results in OPVs

To test the potential of the new fluorinated zinc(II) complexes as electron acceptor, we fabricated bulk heterojunction OPVs in the inverted configuration using P3HT as the electron donor. The best results obtained so far are reported in Table 3. For comparison, we also included results for a typical P3HT:PCBM solar cell. First, we note that best PCEs for Zn(WS3)_2_ are lower than in our previous publication, 2.36% instead of 4.10% [10]. The main difference is that we now get lower *J*_sc_, 5.2 mA/cm^2^ instead of 9.1 mA/cm^2^. While our previously reported results were reproducible at the time, we are no longer able to reproduce them with the new Zn(WS3)_2_ batches, even after extensive purification. We have therefore decided to report the results we now routinely obtain because they are obtained under similar conditions as the new results with the fluorinated compounds.

**Table 3:** Preliminary results for OPVs using P3HT as the donor.

Acceptor	Donor:acceptor ratio	*V*_OC_ [V]	*J*_SC_ [mA/cm^2^]	FF [%]	PCE [%]

Zn(WS3)_2_	1:0.7	0.80	5.16	57	2.36
Zn(L1)_2_	1:1	0.68	5.08	47	1.55
Zn(L2)_2_	1:0.7	0.73	8.31	59	3.04
Zn(L3)_2_	1:0.5	0.73	8.54	60	3.74
Zn(L4)2	1:0.7	0.59	9.29	66	3.26
PCBM	1:0.8	0.59	11.06	61	3.97

Zn(L2–L4)_2_ showed an increase in PCE compared to Zn(WS3)_2_, due to an increase in *J*_SC_. This points to a generally positive effect of fluorination on device performance. The current best performance of 3.74% was obtained with Zn(L3)_2_ with an open circuit voltage (*V*_oc_) of 0.73V, a short-circuit current density (*J*_sc_) of 8.54 mA/cm^2^ and a fill factor (FF) of 60%. While maintaining similar *V*_oc_ and FF values, Zn(L2)_2_ had a lower PCE of 3.04% due to a decrease in *J*_sc_. With a PCE of 3.26%, Zn(L4)_2_ showed the largest *J*_SC_ and FF but a lower *V*_oc_, consistent with its lower LUMO energy level than the other complexes. Zn(L4)_2_ shows the best potential to be paired with other electron donors of lower HOMO energy levels than P3HT. Compared to the P3HT:PCBM cell, the P3HT:Zn(L4)_2_ cell had a similar *V*_oc,_ a higher FF, and a lower *J*_sc_. The lower *J*_sc_ may be due to several factors, including purity, film thickness, morphology and charge recombination differences. Further studies are underway to better understand these results and will be reported separately.

## Conclusion

In conclusion, four fluorinated WS3 derivatives were synthesized and the optical and electrochemical properties of their respective zinc(II) and BF_2_^+^ chelates studied. It was found that the addition of fluorine into the Zn(L1)_2_ and Zn(L4)_2_ complexes raised the oxidation and reduction potentials. Fluorination was found to have little effect on the absorption spectra, both in solution and film. Preliminary results in OPVs suggest that fluorination is generally beneficial for device performance of zinc(II) azadipyrromethene-based acceptors. In particular, Zn(L4)_2_ is the best candidate to directly replace PCBM due to similar energy levels and good performance. More work is underway to better understand the mechanism for the enhancement, and will be published separately.

## Experimental

### Materials

Chalcone (Acros), 4-fluorochalcone (TCI America), 4’-fluorochalcone (TCI America), 4,4’-difluorochalcone (TCI America), 1-ethynyl-4-fluorobenzene (Aldrich), 4-ethynyl-α,α,α-trifluorotoluene (Aldrich), *n*-butyllithium solution (Aldrich), tributyltin chloride (Fisher), tributyl(phenylethynyl)tin (Aldrich), tetrakis(triphenylphosphine)palladium(0) (Aldrich), *n*-iodosuccinimide (abbreviated NIS, Aldrich) were used as received. All other reagents and solvents were used as received unless otherwise specified. Xylenes and tetrahydrofuran were distilled over sodium and benzophenone, degassed and stored under nitrogen. ADP, ADPI_2_, WS3, BF_2_(WS3), and Zn(WS3)_2_ were synthesized and purified according to literature procedures with minor modifications [2,5,9,33–34]. (4-Fluorophenylethynyl)tributyltin (for the synthesis of L3), and [4-(trifluoromethyl)phenyl](tributyltin)acetylene (for the synthesis of L4) were synthesized according to literature procedures with minor modifications and used without purification [35–36].

### Methods

^1^H, ^19^F, and ^13^C NMR spectra were recorded using a 500 MHz Bruker Ascend Avance III HDTM equipped with ProdigyTM ultra-high sensitivity Multinuclear Broadband CryoProbe or a Varian 400 MHz spectrometer in parts-per-million with respect to tetramethylsilane. MALDI–TOF–MS samples were prepared from chloroform solutions in a terthiophene matrix and run on a Bruker Autoflex III MALDI–TOF–TOF–MS. UV–visible absorption and emission spectra were collected in HPLC grade chloroform on a UV-Cary 50 spectrometer and a Cary Eclipse fluorescence spectrometer, respectively. Solutions for films were prepared at 10 mg/mL concentration in HPLC grade chloroform. The solutions were filtered through a 0.45 μm PTFE filter, then spun-cast at 400 rpm for 60 s. Elemental analyses (C, H, and N) were performed under optimum combustion conditions by Robertson Microlit Laboratories. Thermal gravimetric analysis (TGA) was performed on a TA instrument Q500 thermogravimetric analyzer.

Cyclic voltammetry was performed at room temperature using an Auto-Lab-PGSTAT 302N, Exo Chemie potentiostat. Dichloromethane (DCM) was dried over calcium hydride and stored in a nitrogen glove box prior to use. The samples were prepared in a degassed 0.1 M solution of tetra-*n*-butylammonium hexafluorophosphate (TBAPF_6_) in DCM. Ferrocene/ferrocenium was used as an internal standard and was purified prior to use by sublimation. A typical three-electrode configuration was used, with a glassy carbon electrode as the working electrode and two platinum wires used as the counter and pseudoreference electrodes.

Crystals suitable for X-ray diffraction analysis were obtained by the slow diffusion of methanol into a dichloromethane solution. The crystals obtained were dark blue-black in appearance. Single-crystal X-ray diffraction studies were carried out using a Rigaku Rapid II diffractometer using Cu Kα (λ = 1.54178 Å) radiation monochromated using laterally graded multilayer (Goebel) mirror focusing optics. A single crystal was mounted on a Mitegen loop and cooled to 100 K for data collection. Unit cell parameters were measured and data were collected using the Rigaku CrystalClear software [39]. Data were reindexed and integrated using HKL3000, scaled, and corrected for absorption using Scalepack [40]. The space group was assigned and the structure was solved by direct methods using the SHELXTL suite of programs [41–42] and refined by full matrix least squares against *F*^2^ with all reflections using Shelxl 2014 [43] using the graphical interface Shelxle [44]. H atoms attached to carbon atoms were positioned geometrically and constrained to ride on their parent atoms, with carbon hydrogen bond distances of 0.95 Å for aromatic C–H and 0.99 Å for CH_2_ moieties, respectively. *U*_iso_(H) values were set to 1.2 times *U*_eq_(C). A dichloromethane molecule is disordered around a twofold axis.

### Synthesis

**L1-ADP:** 4’-Fluorochalcone (2.01 g, 8.84 mmol) was dissolved in methanol (25 mL) in a round-bottom flask (100 mL) connected with a reflux condenser. Then nitromethane (2.70 g, 44.2 mmol) and diethylamine (3.23 g, 44.2 mmol) were added and refluxed for 24 h. The mixture was cooled to room temperature and then chilled in an ice bath before adding a 1 M HCl solution dropwise (100 mL). The mixture was then extracted 3× with dichloromethane, dried with anhydrous magnesium sulfate, filtered and rotary evaporated to obtain a yellow oil. This oil was then dissolved in butanol (100 mL) in a 500 mL round-bottom flask. Ammonium acetate (24 g) was added to the solution, stirred and heated to reflux. The solution became blue in about 30 min. After continued refluxing for 24 h, about 75% of the butanol was removed by rotary evaporation and the resulting dark blue slurry was filtered to collect the purplish blue solid product after washing with ethanol (200 mL) and vacuum dried overnight (0.484 g, 22.5%). ^1^H NMR (400 MHz, CDCl_3_) δ 8.04–8.02 (d, *J* = 8 Hz, 4H), 7.91–7.88 (t, *J* = 8 Hz, 4H), 7.44–7.41 (t, *J* = 8 Hz, 4H), 7.38–7.35 (t, *J* = 8 Hz, 2H), 7.24–7.20 (t, *J* = 8 Hz, 4H), 7.13 (s, 2H)); ^19^F NMR (470 MHz, CDCl_3_) δ −92.35.

**L2-ADP:** 4-Fluorochalcone (3.29 g, 13.26 mmol) was dissolved in methanol (100 mL) in a round-bottom flask (100 mL) connected with a reflux condenser. Then nitromethane (4.32 g, 70.8 mmol) and diethylamine (5.66 g, 77.4 mmol) were added and heated to reflux for 24 h. The mixture was cooled to room temperature and then chilled on an ice bath before adding 1 M HCl solution drop-wise (100 mL). The mixture was then extracted 3× with dichloromethane, dried with anhydrous magnesium sulfate, filtered and rotary evaporated to obtain a yellow oil. This oil was then dissolved in 1-butanol (200 mL) in a 500 mL round-bottom flask. Ammonium acetate (37.6 g) was added to the solution and stirred and heated to reflux. After continued refluxing for 24 h, the 1-butanol was removed by rotary evaporation and the resulting solid was suspended in ethanol, filtered, and washed with ethanol (100 mL), and hexanes (50 mL). The solid was collected and vacuum dried overnight (0.919 g, 26%). ^1^H NMR (500 MHz, CDCl_3_) δ 8.00 (dd, *J* = 8 Hz, 2H), 7.96 (d, *J* = 8 Hz, 2H), 7.55 (t, *J* = 7.5 Hz, 4H), 7.48 (t, *J* = 7.5 Hz, 2H), 7.16 (s, 2H), 7.12 (t, *J* = 7 Hz, 4H); ^19^F NMR (470 MHz, CDCl_3_) δ −113.37.

**L1-ADPI_2_**: A similar procedure was used as for ADPI_2_ (0.550 g, 75%) [33]. ^19^F NMR (470 MHz, CDCl_3_) δ −92.35.

**L2-ADPI_2_**: A similar procedure was used as for ADPI_2_ (0.998 g, 82%) [33]. MALDI–TOF–MS *m*/*z*: calcd for C_32_H_19_F_2_I_2_N_3_, 736.96; found, 735.85.

**L1:** Tributyl(phenylethynyl)tin (318 mg, 0.813 mmol) and L1-ADPI_2_ (200 mg, 0.271 mmol) was taken into a Schlenk flask (50 mL) which was evacuated and refilled with N_2_ three times. Dry chlorobenzene (15 mL) was added to the flask using a syringe and stirred and N_2_ was bubbled through for 10 min. Then Pd(PPh_3_)_4_ (0.094 g, 10 mmol %) was added inside a glove box. The mixture was heated at 90 °C for 48 h under N_2_. After cooling to room temperature, the mixture was dissolved in dichloromethane (500 mL) and passed through a Celite plug. The filtrate was concentrated using rotary evaporation of dichloromethane and poured into methanol (300 mL) to precipitate. The precipitate was filtered and washed with methanol and ether. After drying under vacuum overnight, the product was obtained as a dark blue solid (0.145 g, 78%). ^19^F NMR (470 MHz, CDCl_3_, δ) −92.35.

**L2**: Tributyl(phenylethynyl)tin (0.928 g, 2.373 mmol) and L2-ADPI_2_ (0.500 g, 0.678 mmol) was taken into a Schlenk flask (100 mL), which was evacuated and refilled with N_2_ three times. Distilled xylenes (50 mL) were added to the flask using a syringe and placed under slight vacuum. Then Pd(PPh_3_)_4_ (0.110 g, 14 mmol %) was added inside a glove box. The mixture was heated at 125 °C for 24 h under N_2_. After cooling to room temperature, the mixture was concentrated to a solid using rotary evaporation. The mixture was then cooled on dry ice and triturated with cold methanol (125 mL), filtered, and then washed with cold diethyl ether (125 mL). After drying under vacuum overnight, the product was obtained as a dark blue solid (0.441 g, 94%). MALDI–TOF–MS *m*/*z*: calcd. for C_48_H_29_F_2_N_3_, 685.23; found, 684.06.

**L3**: (4-Fluorophenylethynyl)tributyltin (0.612 g, 1.495 mmol) and ADPI_2_ (0.300 g, 0.427 mmol) was taken into a Schlenk flask (100 mL), which was evacuated and refilled with N_2_ three times. Distilled xylenes (50 mL) were added to the flask using a syringe and placed under slight vacuum. Then Pd(PPh_3_)_4_ (0.049 g, 10 mmol %) was added inside a glove box. The mixture was heated at 125 °C for 48 h under N_2_. After 48 h, additional (4-fluorophenylethynyl)tributyltin (0.612 g, 1.495 mmol) and Pd(PPh_3_)_4_ (0.049 g, 10 mmol %) were added in one shot. The reaction mixture was allowed to react an additional 48 h at 125 °C under N_2_. After cooling to room temperature, the mixture was concentrated using rotary evaporation. The mixture was then cooled on dry ice and triturated with cold methanol (125 mL), filtered, and then washed with cold diethyl ether (125 mL). After drying under vacuum overnight, the product was obtained as a dark blue solid (0.206 g, 70%). MALDI–TOF–MS *m*/*z*: calcd. for C_48_H_29_F_2_N_3_, 685.23; found, 684.39.

**L4**: [4-(Trifluoromethyl)phenyl](tributyltin)acetylene (0.686 g, 1.495 mmol) and ADPI_2_ (0.313 g, 0.445 mmol) was taken into a Schlenk flask (100 mL), which was evacuated and refilled with N_2_ three times. Distilled xylenes (50 mL) were added to the flask using a syringe and placed under slight vacuum. Then Pd(PPh_3_)_4_ (0.049g, 10 mmol %) was added inside a glove box. The mixture was heated at 125 °C for 48 h under N_2_. After 48 h, additional [4-(trifluoromethyl)phenyl](tributyltin)acetylene (0.686 g, 1.495 mmol) and Pd(PPh_3_)_4_ (0.049g, 10 mmol %) were added in one shot. The reaction mixture was allowed to react an additional 48 h at 125 °C under N_2_. After cooling to room temperature, the mixture was concentrated using rotary evaporation. The mixture was then cooled on dry ice and triturated with cold methanol (125 mL), filtered, and then washed with cold diethyl ether (125 mL). After drying under vacuum overnight, the product was obtained as a dark blue solid (0.301 g, 89%). MALDI–TOF–MS *m*/*z*: calcd. for C_50_H_29_F_6_N_3_, 785.79; found, 783.94

**BF_2_(L1)**: It was synthesized using a similar procedure as for BF_2_(WS3)[34]. L1 (0.042 g, 0.1 mmol) was added to a dry round bottom flask (100 mL), sealed, flushed with nitrogen and charged with anhydrous DCM (25 mL). Diisopropylethylamine (0.2 mL) was added via syringe followed immediately by the addition of trifluoroboron etherate (0.4 mL) by syringe. The reaction mixture was stirred for 16 h at room temperature under N_2_. The solution was washed with distilled water (≈50 mL × 3) and dried over anhydrous MgSO_4_ prior to concentration by rotary evaporation. The crude solid was further purified by column chromatography on silica gel using a 1:1 ratio of DCM/hexanes (v/v). The final pure product was obtained as a dark blue solid after removal of solvents (0.047 g, 74%) ^1^H NMR (500 MHz, CD_2_Cl_2_, δ) 8.26–8.24 (d, *J* = 10 Hz, 4H), 8.08–8.05 (t, *J* = 10 Hz, 4H), 7.56–7.49 (m, 6H), 7.34 (s, 10H), 7.28–7.24 (t, *J* = 10 Hz, 5H); ^19^F NMR (470 MHz, CDCl_3_, δ) −108.30–108.40 (m), −131–131.5 (dd); MALDI–TOF–MS *m*/*z*: calcd. for C_48_H_28_BF_4_N_3_, 733.23; found, 731.86; anal. calcd for: C, 78.59; H, 3.85; N, 5.73; found: C, 78.56; H, 4.00; N, 5.45.

**BF_2_(L3)**: It was synthesized using a similar procedure as for BF_2_(L1) (0.039 g, 73%). ^1^H NMR (500 MHz, CDCl_3_) δ 8.23 (m, 4H), 8.01 (m, 4H), 7.51 (m, 13H), 7.28 (m, 5H), 7.00 (t, *J* = 7 Hz, 4H); ^19^F NMR (470 MHz, CDCl_3_) δ −109.30–110.40 (m), −131–131.1 (dd); MALDI–TOF–MS *m*/*z*: calcd. for C_48_H_28_BF_4_N_3_, 733.23; found, 731.94; anal. calcd for: C, 78.59; H, 3.85; N, 5.73; found: C, 78.41; H, 3.62; N, 5.98.

**BF_2_(L4)**: It was synthesized using a similar procedure than that of BF_2_(L1) (0.064 g, 59%). ^1^H NMR (500 MHz, CDCl_3_) δ 8.22 (m, 4H), 8.03 (m, 4H), 7.54 (m, 14H), 7.4 (m, 4H); ^19^F NMR (470 MHz, CDCl_3_, δ) −62.9 (s), −130–131.1 (dd); MALDI–TOF–MS *m*/*z*: calcd. for C_50_H_28_BF_8_N_3_, 833.22; found, 831.90; anal. calcd for: C, 72.04; H, 3.39; N, 5.04; found: C, 74.24; H, 3.25; N, 5.30.

**Zn(L1)_2_**: In a 100 mL three-necked flask equipped with a reflux condenser, L1 (100 mg, 0.146 mmol) was dissolved in anhydrous tetrahydrofuran (10 mL) under N_2_. To this dark blue solution, anhydrous NaH (4.00 mg, 0.161 mmol) was added and the mixture was heated to 60 °C. The solution turned to bright blue. After 24 h of heating, anhydrous ZnCl_2_ (11.5 mg, 0.084 mmol) was added and heating was continued for another 24 h. The mixture turned back to dark blue. Then the mixture was cooled to room temperature and dissolved in dichloromethane (200 mL). This solution was then passed through a Celite plug and the filtrate was collected. The crude product was obtained by rotary evaporation of dichloromethane, and purified by flash chromatography using a dichloromethane/hexane mixture as the eluent (starting with 80% hexane and gradually decreasing the amount to 60%). The final pure product was obtained as a dark blue solid after removal of solvents (0.091 g, 87%). ^1^H NMR (400 MHz, CD_2_Cl_2_) δ 7.98–7.97 (d, *J* = 4 Hz, 8H), 7.84–7.81 (dd, *J* = 8 Hz, *J* = 4 Hz, 8H), 7.54–7.46 (m, 12H), 7.39–7.36 (m, 8H), 7.35–7.32 (m, 12H), 7.04–7.00 (t, *J* = 8 Hz, 8H); ^19^F NMR (470 MHz, CDCl_3_) δ −110.36 (s); MALDI–TOF–MS *m*/*z*: calcd for C_96_H_56_F_4_N_6_Zn, 1433.38; found, 1431.36. anal. calcd for: C, 80.36; H, 3.93; N, 5.86; found: C, 80.58; H, 4.08; N, 5.89.

**Zn(L2)_2_**: In a 100 mL three-necked flask L2 (0.229 g, 0.334 mmol) was dissolved in anhydrous tetrahydrofuran (20 mL) under N_2_. To this dark blue solution, anhydrous NaH (0.008 g, 0.401 mmol) was added and the mixture was heated to 60 °C. The solution turned bright blue. After 16 h of heating, anhydrous ZnCl_2_ (0.023 g, 0.168 mmol) was added and heating was continued for another 16 h. The crude product was obtained by rotary evaporation of dichloromethane and purified by flash chromatography using a dichloromethane/hexane mixture as the eluent (started with 80% hexane and gradually decreasing to 60%). The final pure product was obtained as a dark blue solid after removal of solvents (0.167 g, 70%). ^1^H NMR (400 MHz, CD_2_Cl_2_, δ) 7.94 (dd, *J* = 8 Hz, 8H), 7.78 (m, 8H), 7.36 (m, 8H), 7.30 (m, 12H), 7.22 (m, 12H), 7.17 (t, *J* = 7 Hz, 8H); ^19^F NMR (470 MHz, CDCl_3)_ δ −112.77 (s). MALDI–TOF–MS *m*/*z*: calcd for C_96_H_56_F_4_N_6_Zn, 1433.38; found, 1432.46; anal. calcd for: C, 80.36; H, 3.93; N, 5.86; found: C, 80.19; H, 4.04; N, 5.78.

**Zn(L3)_2_**: In a 100 mL three-necked flask L3 (0.166 g, 0.242 mmol) was dissolved in anhydrous tetrahydrofuran (15 mL) under N_2_. To this dark blue solution, anhydrous NaH (0.006 g, 0.266 mmol) was added and the mixture was heated to 60 °C. The solution became bright blue. After 16 h of heating, anhydrous ZnCl_2_ (0.016 g, 0.121 mmol) was added and heating was continued for another 16 h. The crude product was obtained by rotary evaporation of dichloromethane and purified by flash chromatography using a dichloromethane/hexane mixture as the eluent (starting with 80% hexane and gradually decreasing to 60%). The final pure product was obtained as a dark blue solid after removal of solvents (0.119 g, 69%). ^1^H NMR (400 MHz, CD_2_Cl_2_, δ) 7.94 (d, *J* = 8 Hz, 8H), 7.78 (d, *J* = 8 Hz, 8H), 7.48 (m, 12H), 7.34 (m, 8H), 7.01 (t, *J* = 7 Hz, 8H); ^19^F NMR (470 MHz, CDCl_3_) δ −111.77 (s). MALDI–TOF–MS *m*/*z*: calcd for C_96_H_56_F_4_N_6_Zn, 1433.38; found, 1432.80; anal. calcd for: C, 80.36; H, 3.93; N, 5.86; found: C, 80.13; H, 4.09; N, 5.69.

**Zn(L4)_2_**: In a 100 mL three-necked flask L4 (0.346 g, 0.440 mmol) was dissolved in anhydrous tetrahydrofuran (20 mL) under N_2_. To this dark blue solution, anhydrous NaH (0.012 g, 0.484 mmol) was added and the mixture was heated to 60 °C. The solution turned into a bright blue solution. After 16 h of heating, anhydrous ZnCl_2_ (0.030 g, 0.220 mmol) was added and heating was continued for another 16 h. The crude product was obtained by rotary evaporation of dichloromethane and purified by flash chromatography using dichloromethane/hexane mixture as the eluent (started with 80% hexane and gradually decreasing to 60%). The final pure product was obtained as a dark blue solid after removal of solvents (0.195 g, 54%). ^1^H NMR (400 MHz, CD_2_Cl_2_) δ 7.90 (d, *J* = 8 Hz, 8H), 7.76 (m, 8H), 7.63 (d, *J* = 7 Hz, 8H), 7.46–7.44 (dd, *J* = 8 Hz, 20H); ^19^F NMR (470 MHz, CDCl_3_) δ −63.07 (s). MALDI–TOF–MS *m*/*z*: calcd for C_96_H_56_F_4_N_6_Zn, 1633.37; found, 1629.75; anal. calcd for: C, 73.46; H, 3.45; N, 5.14; found: C, 73.42; H, 3.69; N, 5.06.

### Organic solar cells

Photovoltaic properties were studied using the inverted configuration: ITO/ZnO/P3HT:Acceptor/MoO_3_/Ag. ITO-coated glass (*R* = 15 Ω/sq) substrates were cleaned stepwise in each of the following under ultra-sonication for 15 minutes: detergent, deionized water, acetone, and isopropanol. From a 0.25 M ZnO precursor solution, the ZnO layer was spun coat. For all of the devices fabricated, the total concentration of the active layer was 20 mg/mL with varying donor-to-acceptor ratios (see Table 3). While in an oxygen and moisture-free environment, the photoactive layer was spun coat at 1000 rpm for 40 s and 2000 rpm for 2 s. The substrates were annealed at 120 °C for 30 min prior to top electrode deposition. The P3HT:PCBM devices had a total concentration of 40 mg/mL, 1:0.8 donor-to-acceptor ratio, were spun coat at 800 rpm for 40 s and 2000 rpm for 2 s and annealed at 120 °C for 15 min. Molybdenum oxide (10 nm) and silver (80 nm) were thermally evaporated in sequence under a vacuum pressure of ≈3 × 10^−6^ Torr using an Angstrom Engineering Evovac thermal evaporator. The devices were characterized using a Oriel Sol2A solar simulator and a Keithley 2400 SourceMeter. The active area of each solar cell is 0.20 cm^2^.

## Supporting Information

File 1 NMR and MS data.

File 2 Crystal structure of Zn(L2)_2_.
