# Supplementary material for: Synthesis and characterization of fluorinated azadipyrromethene complexes as acceptors for organic photovoltaics
Source: Beilstein J Org Chem. 2016 Aug 29;12:1925–38. doi: 10.3762/bjoc.12.182 (PMC5082603; doi:10.3762/bjoc.12.182)
Supplement: File 1 — NMR and MS data. [file Beilstein_J_Org_Chem-12-1925-s001.pdf]

Supporting Information

for

Synthesis and characterization of fluorinated azadipyrromethene complexes as acceptors for organic photovoltaics

Forrest S. Etheridge[1], Roshan J. Fernando[1], Sandra Pejić[1], Matthias Zeller[2] and Geneviève Sauvé*[,1]

Address: [1]Department of Chemistry, Case Western Reserve University, Cleveland, Ohio 44106, United States and [2]Department of Chemistry, Purdue University, West Lafayette, Indiana 47907, United States

Email: Geneviève Sauvé* - genevieve.sauve@case.edu

*Corresponding author

NMR and MS data

This includes all ^{1}H, ^{13}C, and ^{19}F NMR as well as MALDI-TOF MS spectra for the compounds in order of appearance in the experimental section in the main text.

L1-ADP

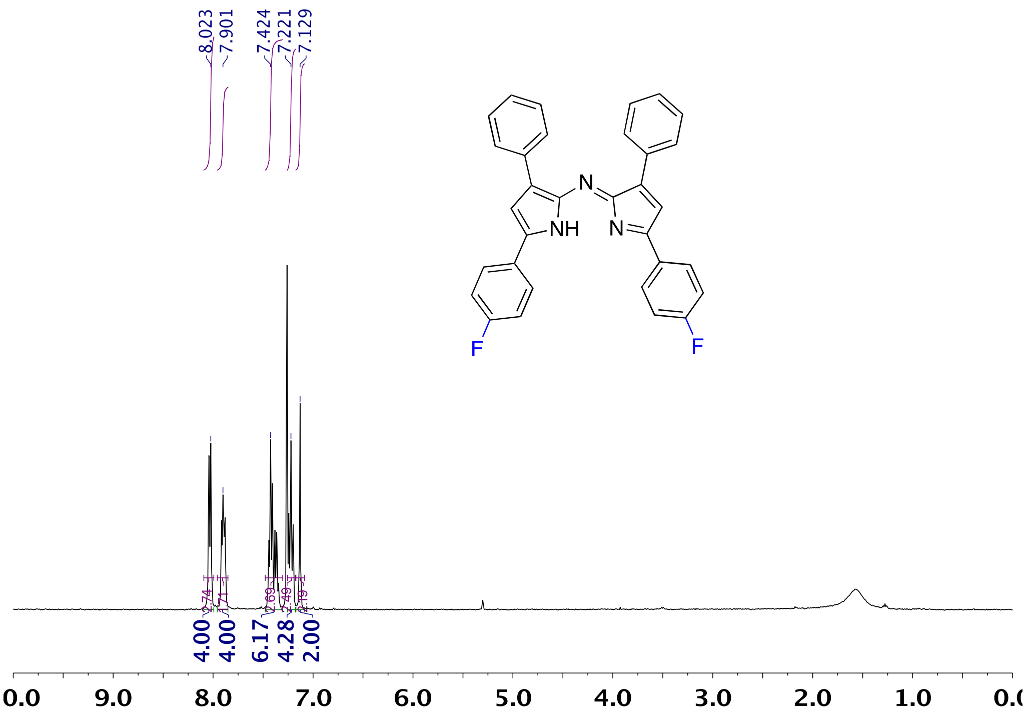

Figure S1 ^{1}H NMR of L1-ADP

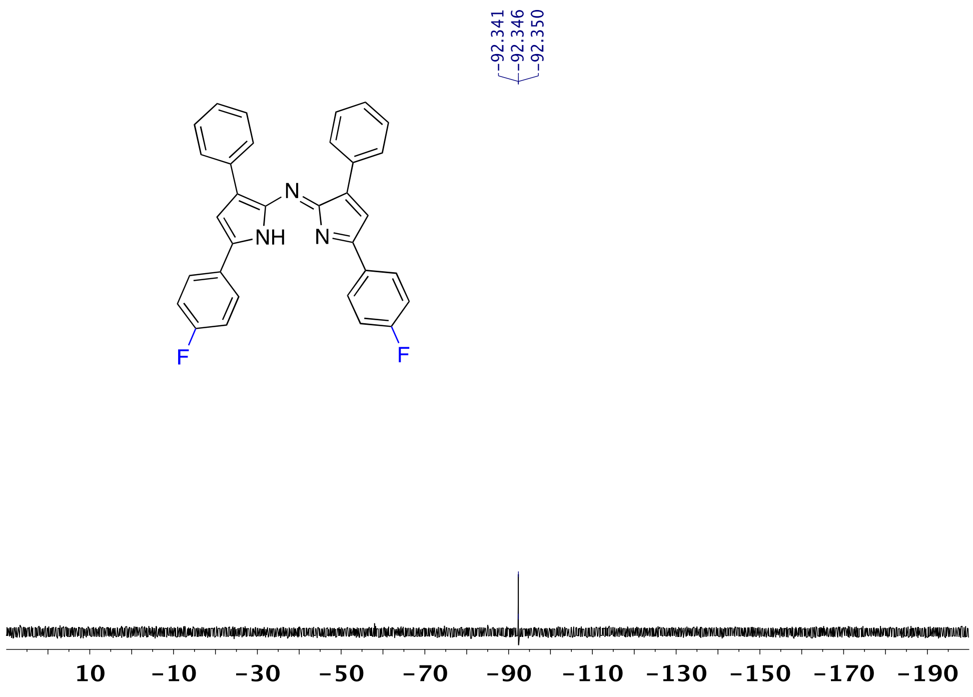

Figure S2 ^{19}F NMR of L1-ADP

L2-ADP

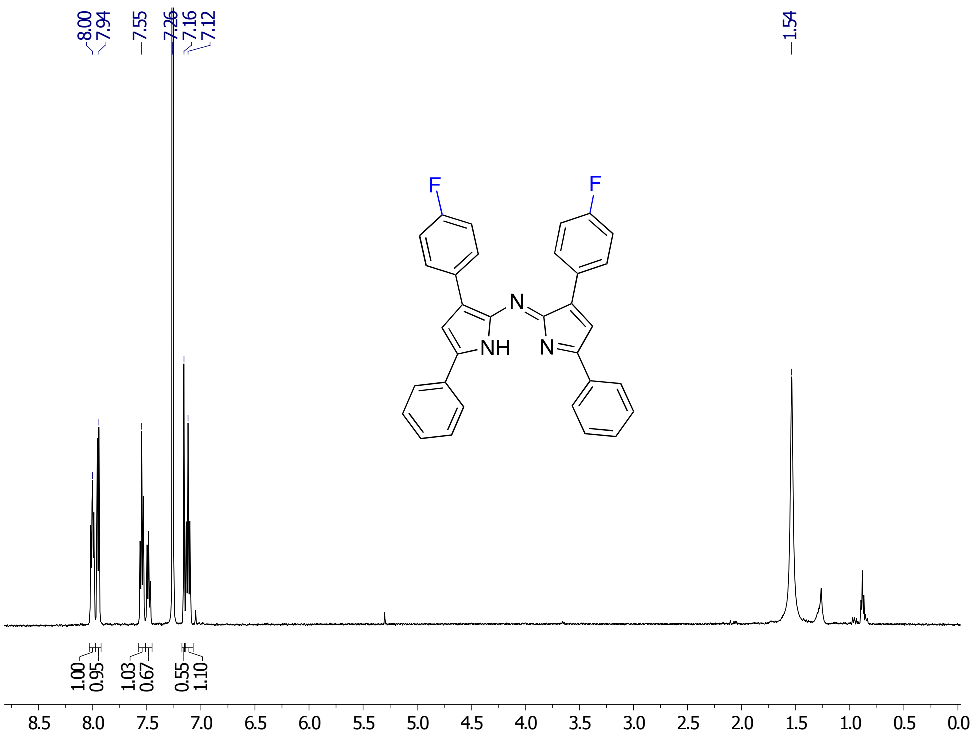

Figure S3 ^{1}H NMR of L2-ADP

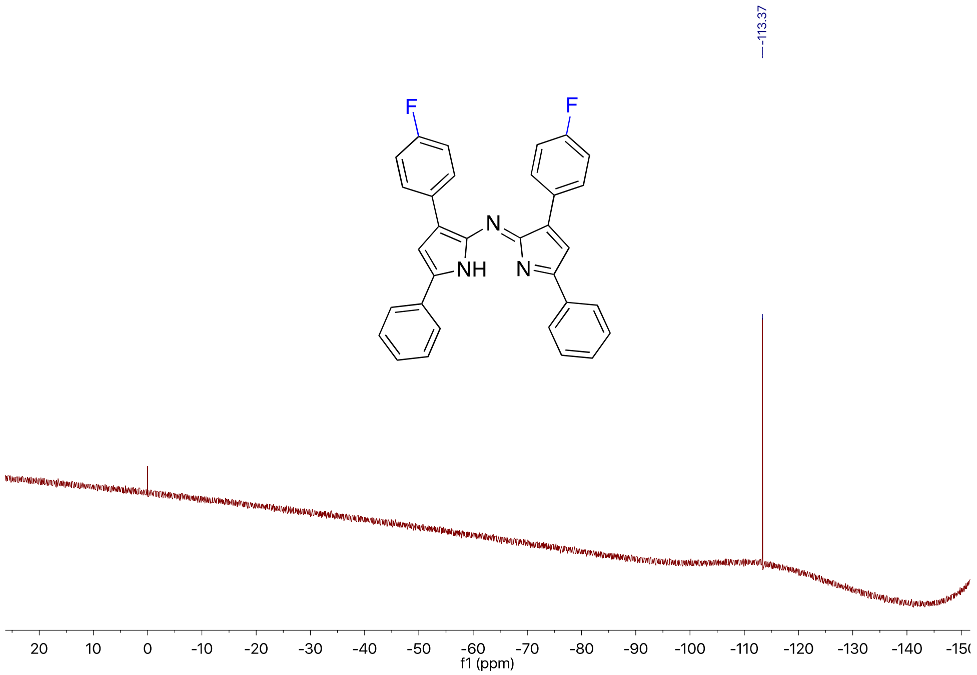

Figure S4 ^{19}F NMR of L2-ADP

L1-ADPI$_2$

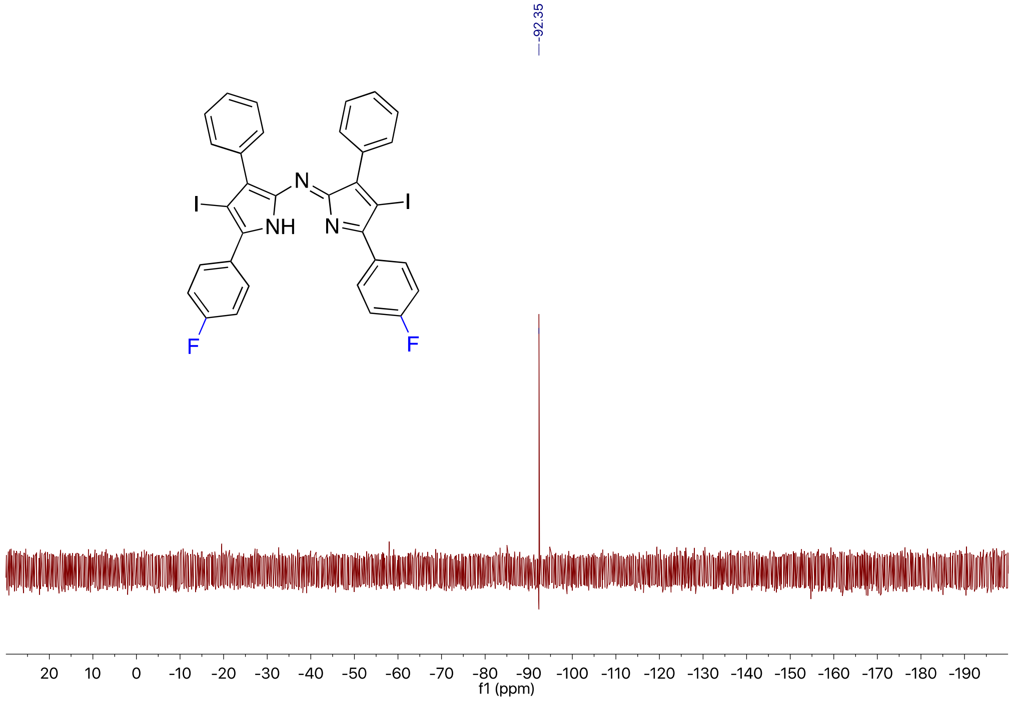

Figure S5 ^{19}F NMR of L1-ADPI$_2$

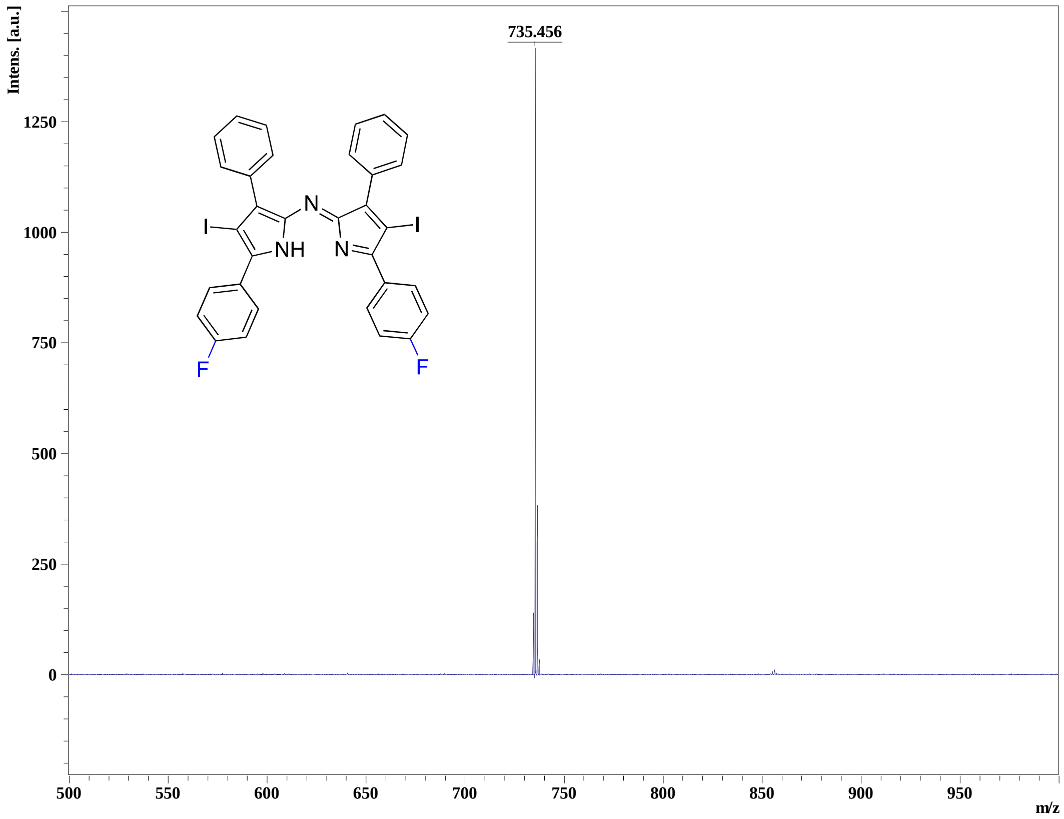

Figure S6 MALDI-TOF MS of L1-ADPI$_2$

L2-ADPI$_2$

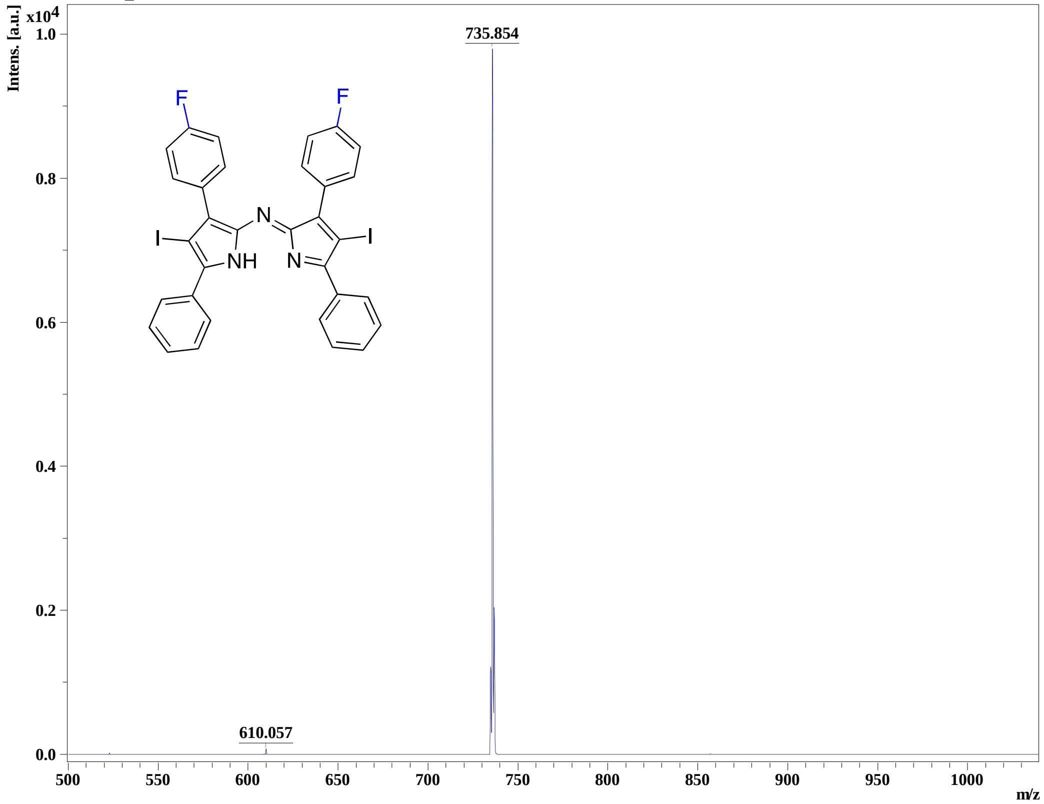

Figure S7 MALDI-TOF MS of L2-ADPI$_2$

L1

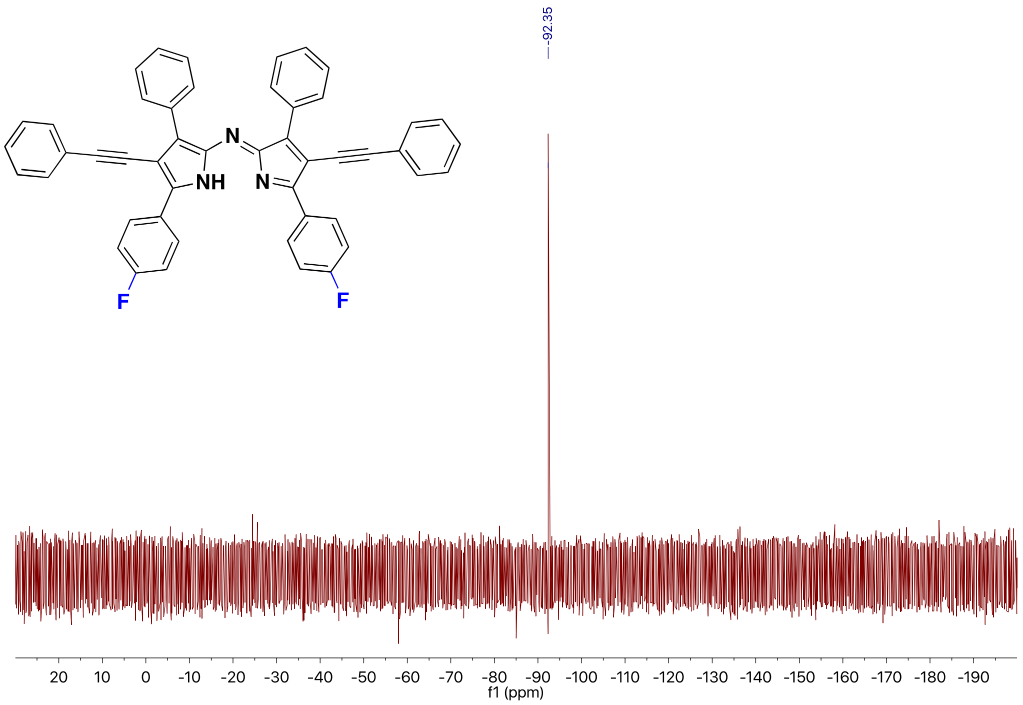

Figure S8 ^{19}F NMR of L1

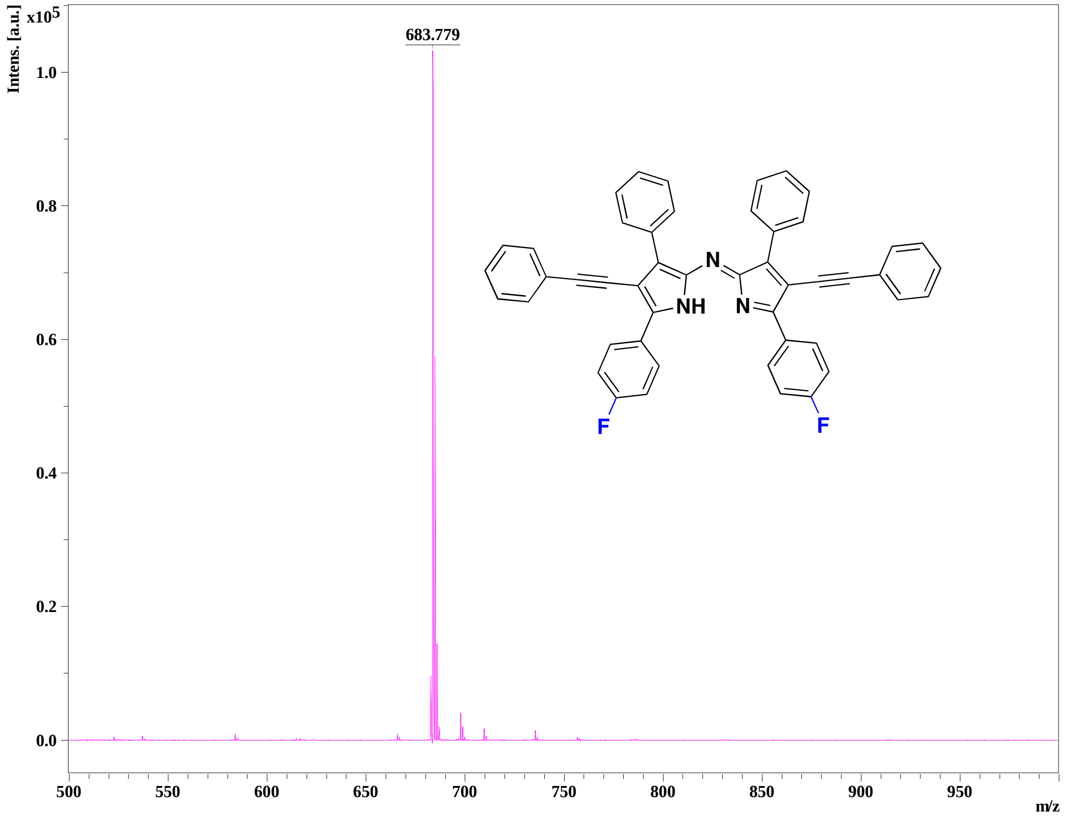

Figure S9 MALDI-TOF MS of L1

L2

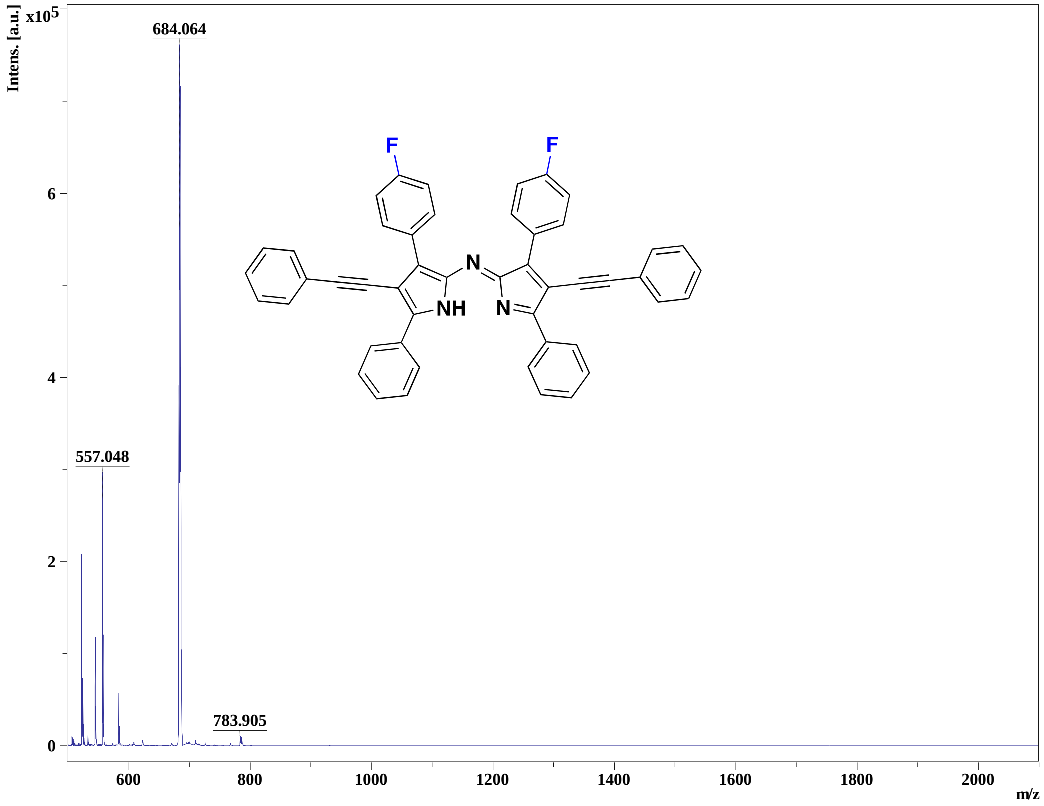

Figure S10 MALDI-TOF MS of L2

L3

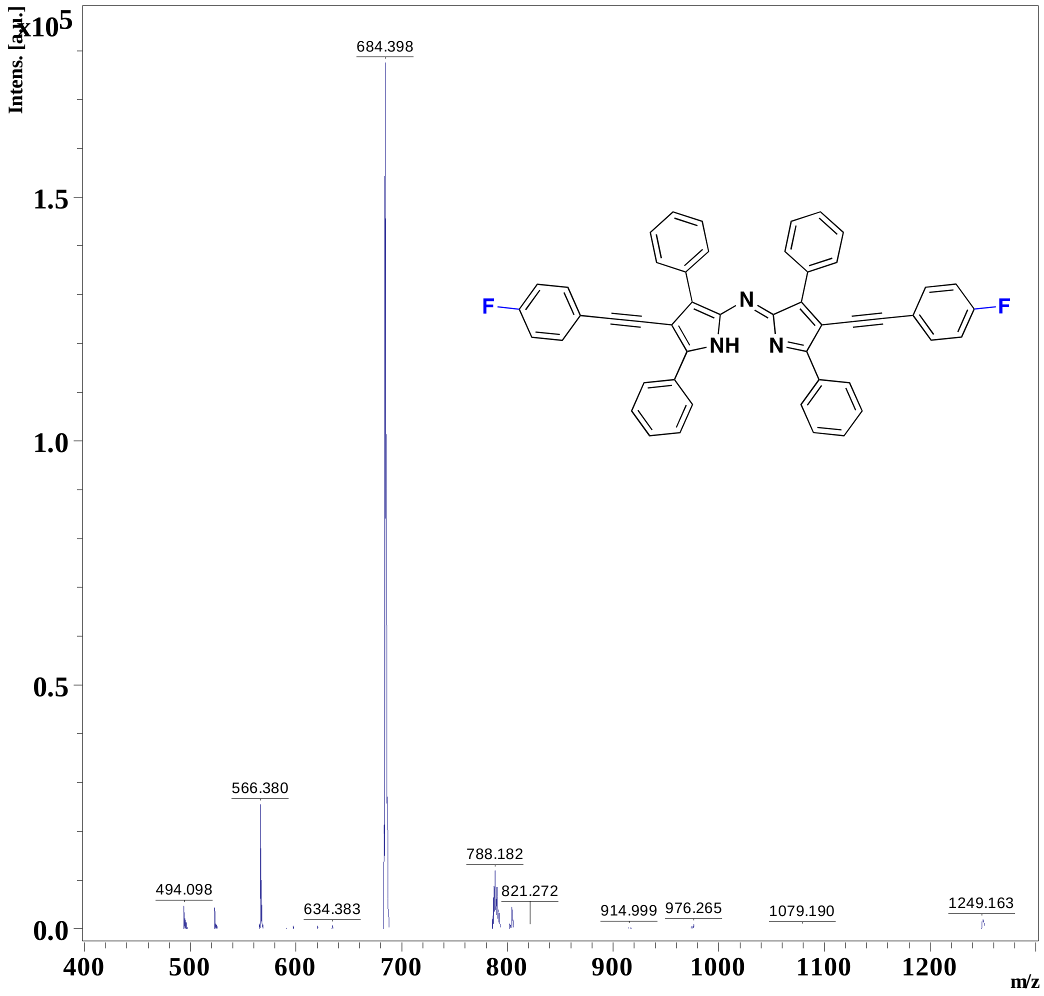

Figure S11 MALDI-TOF MS of L3

L4

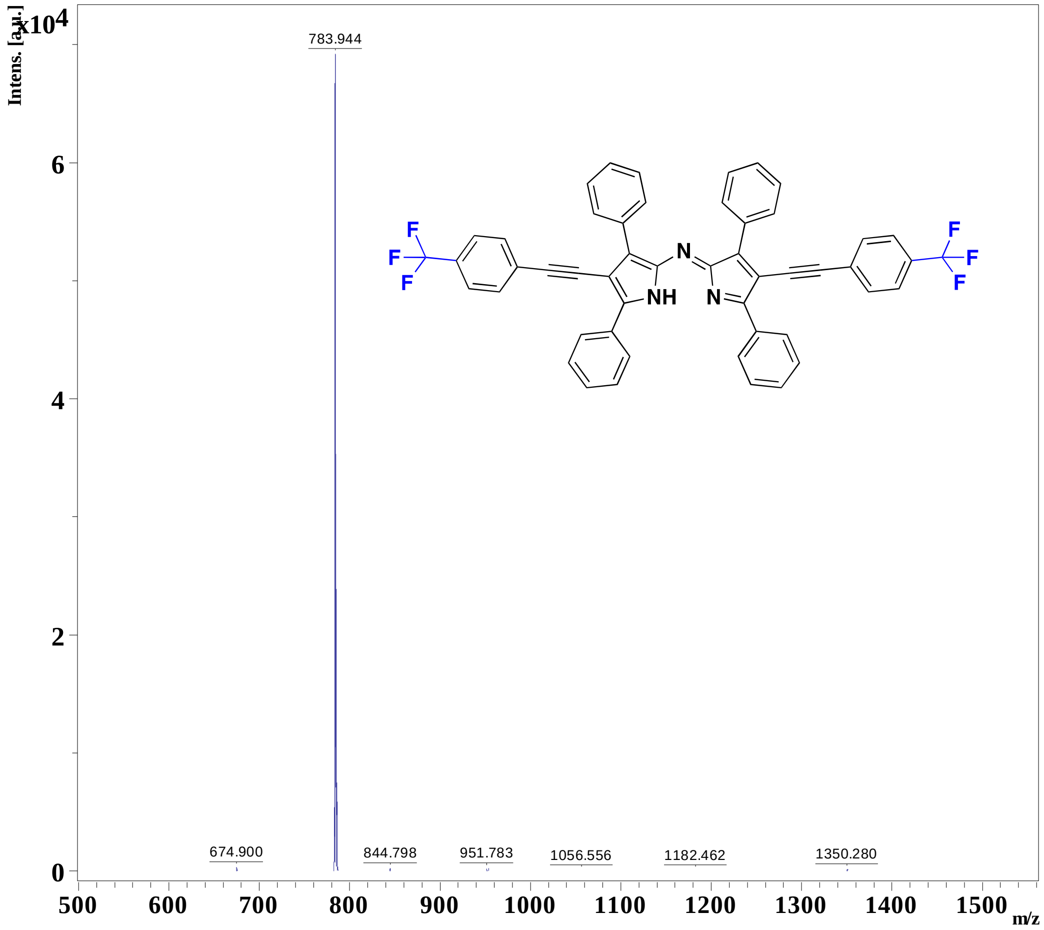

Figure S12 MALDI-TOF MS of L4

BF_2(L1)

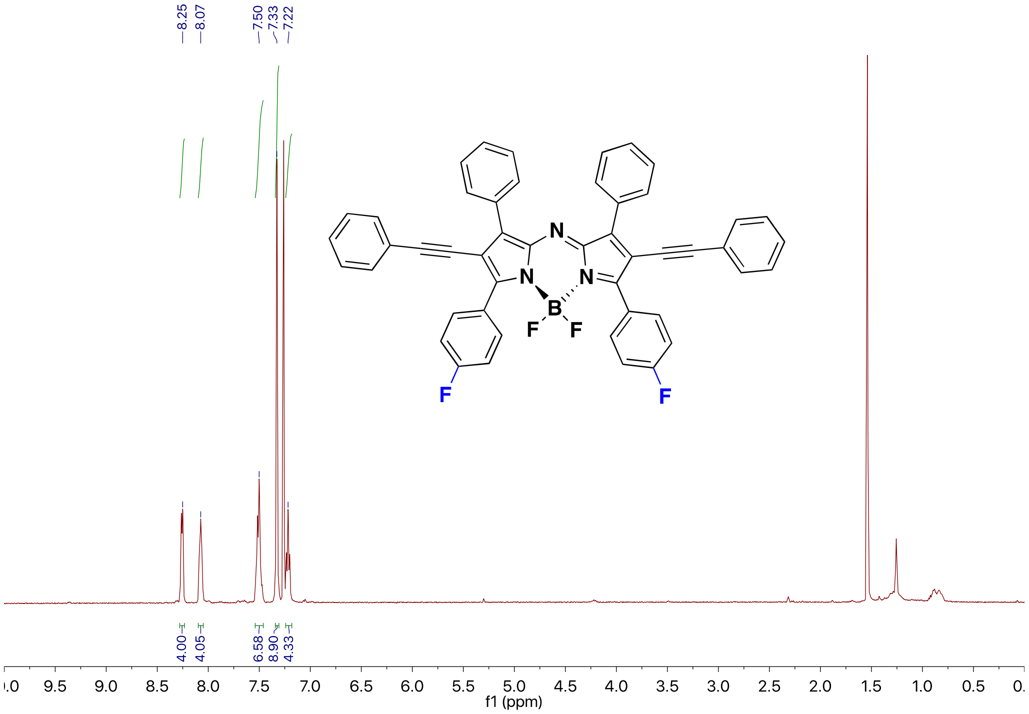

Figure S13 ^{1}H NMR of BF_2(L1)

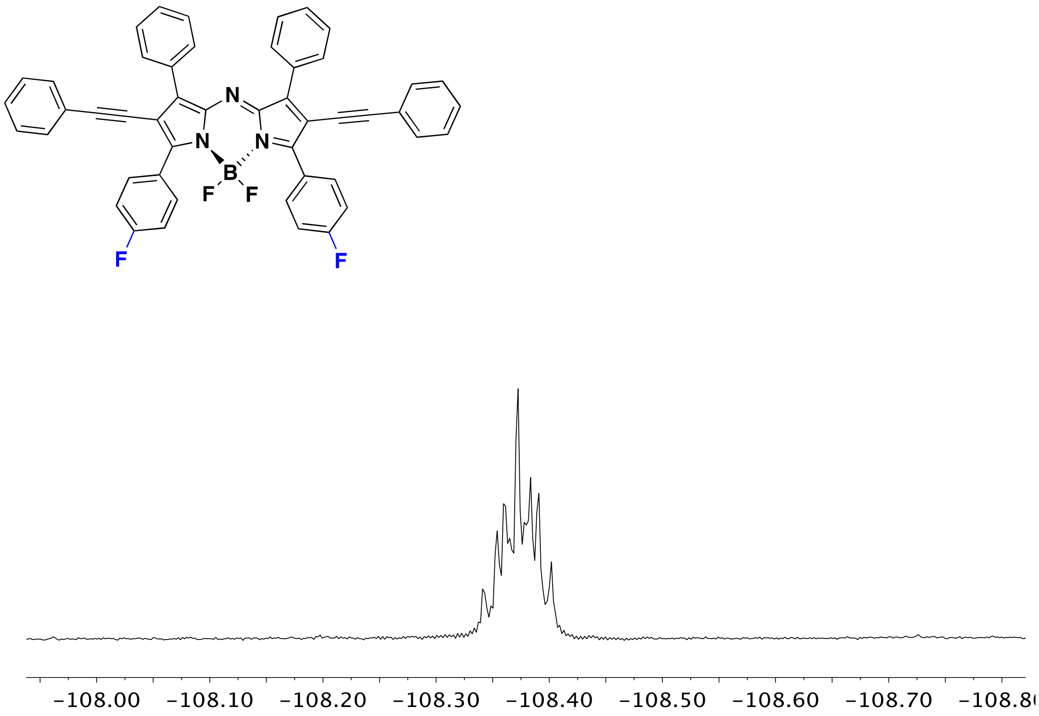

Figure S14 ^{19}F NMR of BF_2(L1)

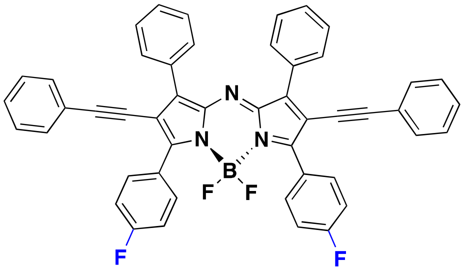

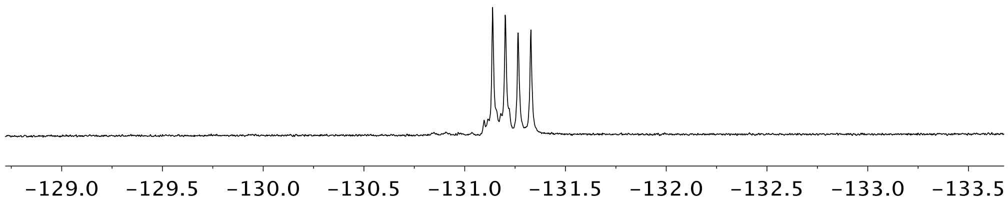

Figure S15 ^{19}F NMR of BF_2(L1), BF_2 peaks

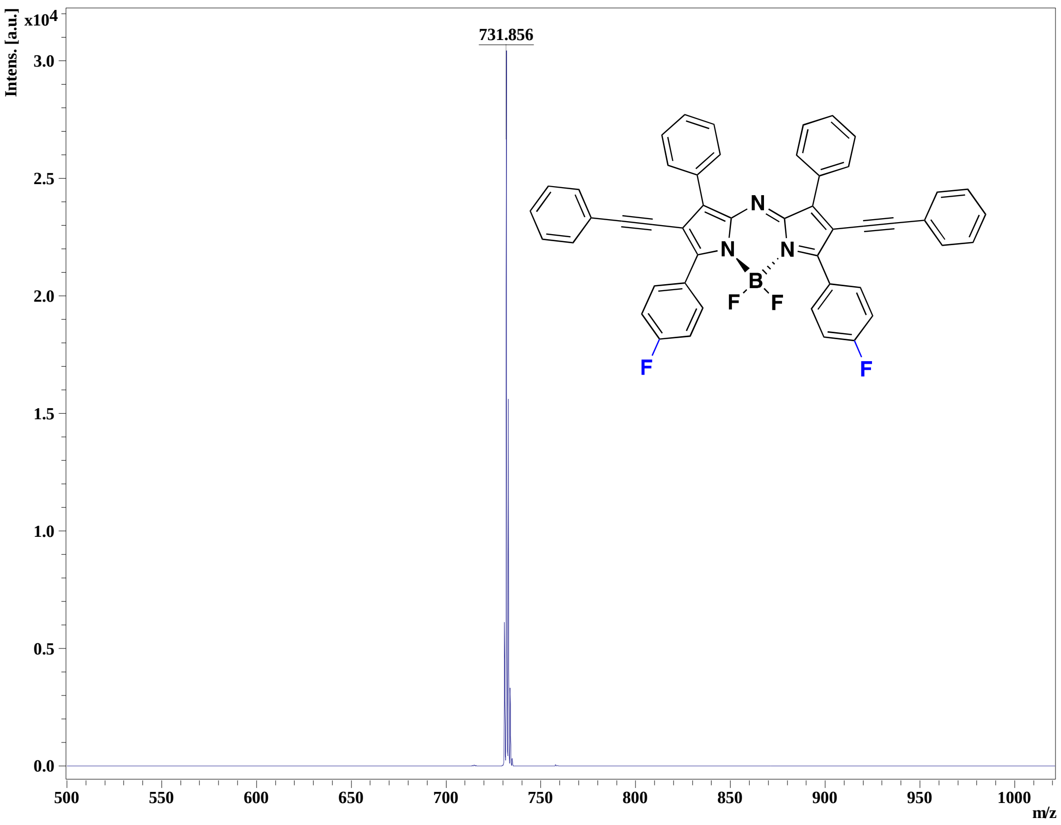

Figure S16 MALDI-TOF MS of BF_2(L1)

BF_2(L3)

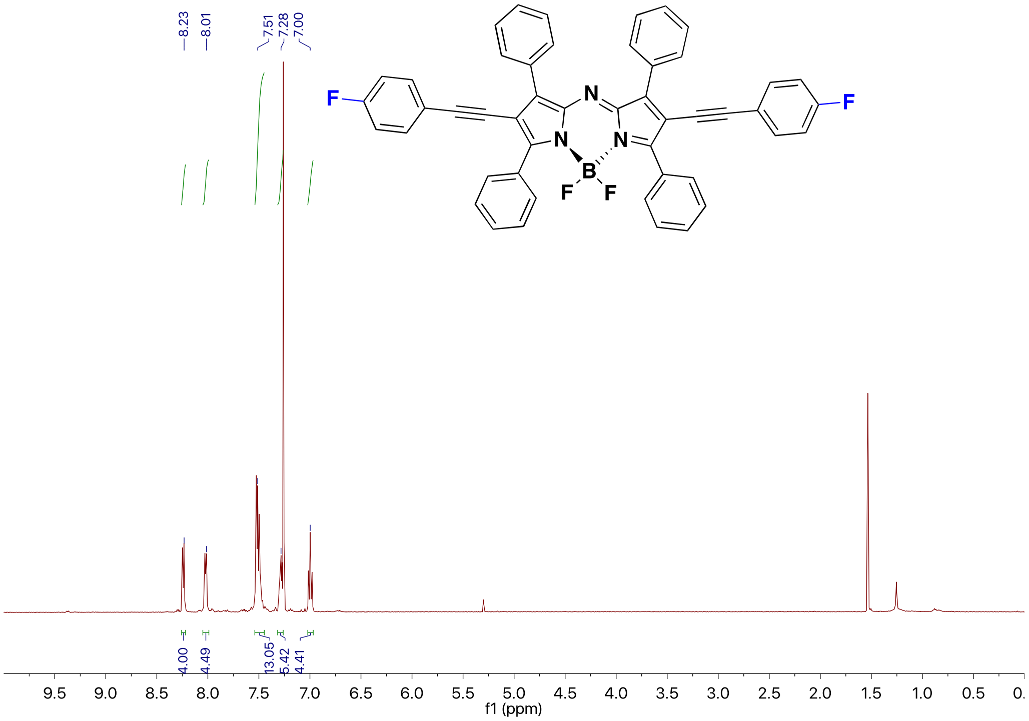

Figure S17 ^{1}H NMR of BF_2(L3)

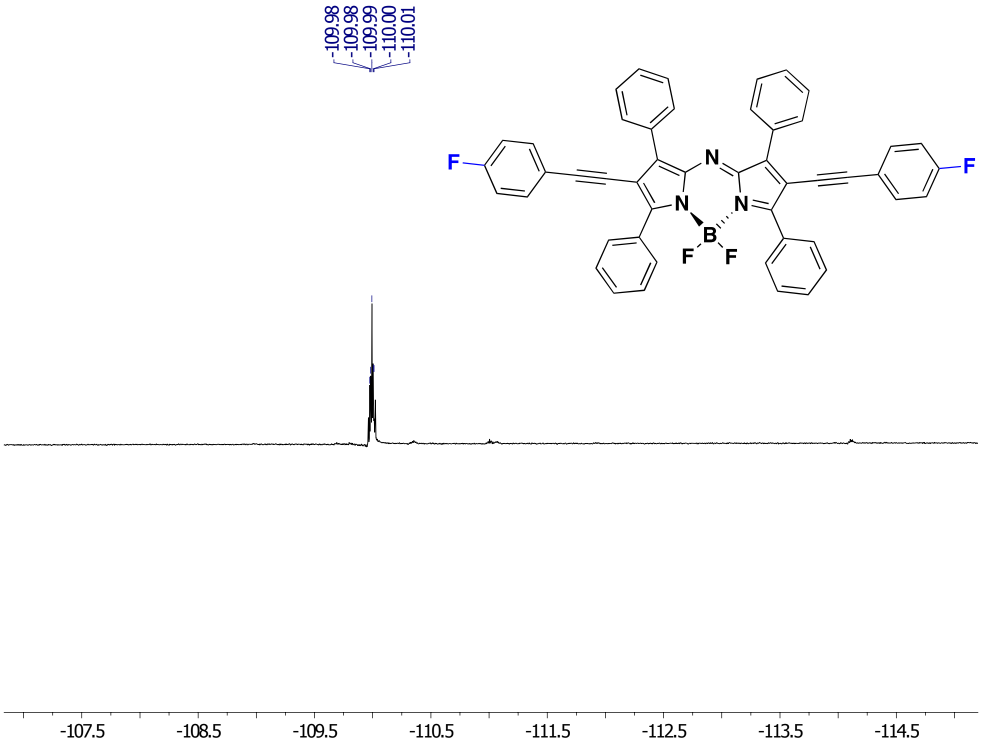

Figure S18 ^{19}F NMR of BF_2(L3)

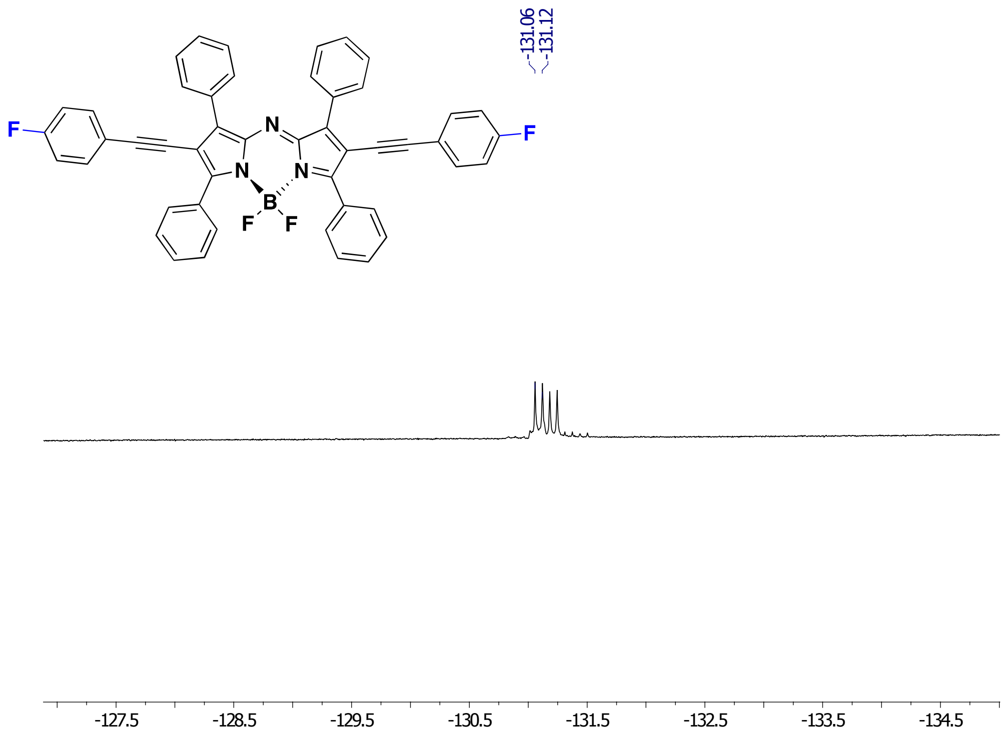

Figure S19 ^{19}F NMR of BF_2(L3), BF_2 peaks

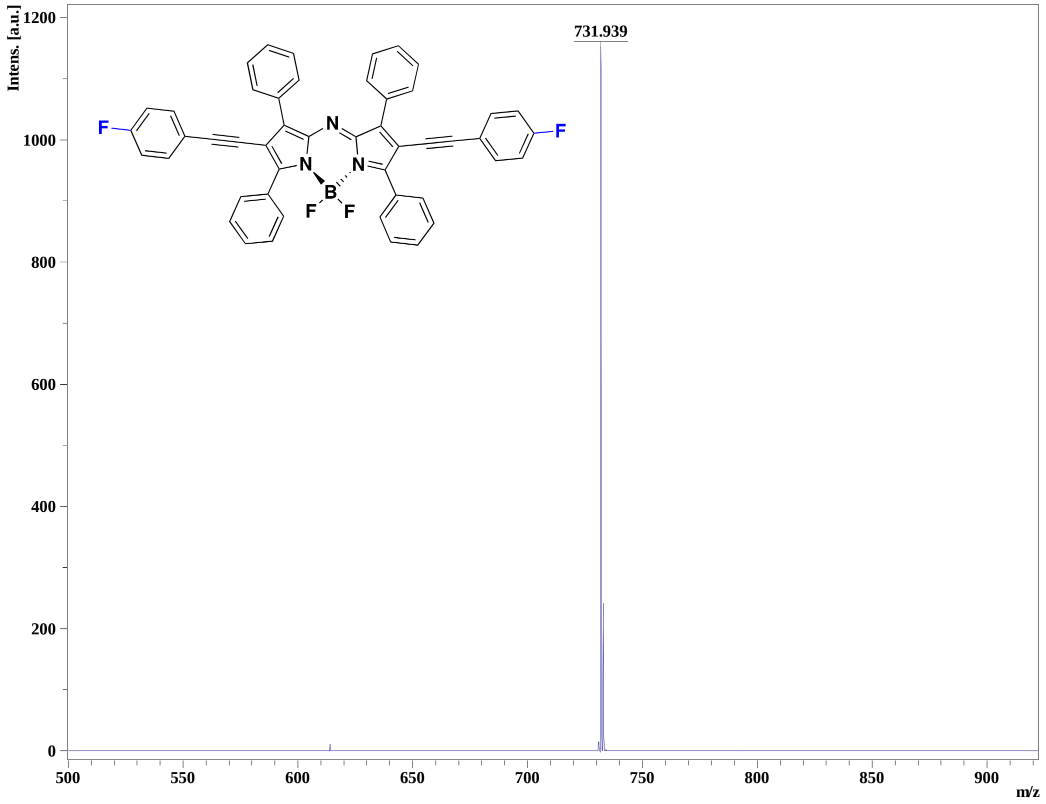

Figure S20 MALDI-TOF MS of BF_2(L3)

BF_2(L4)

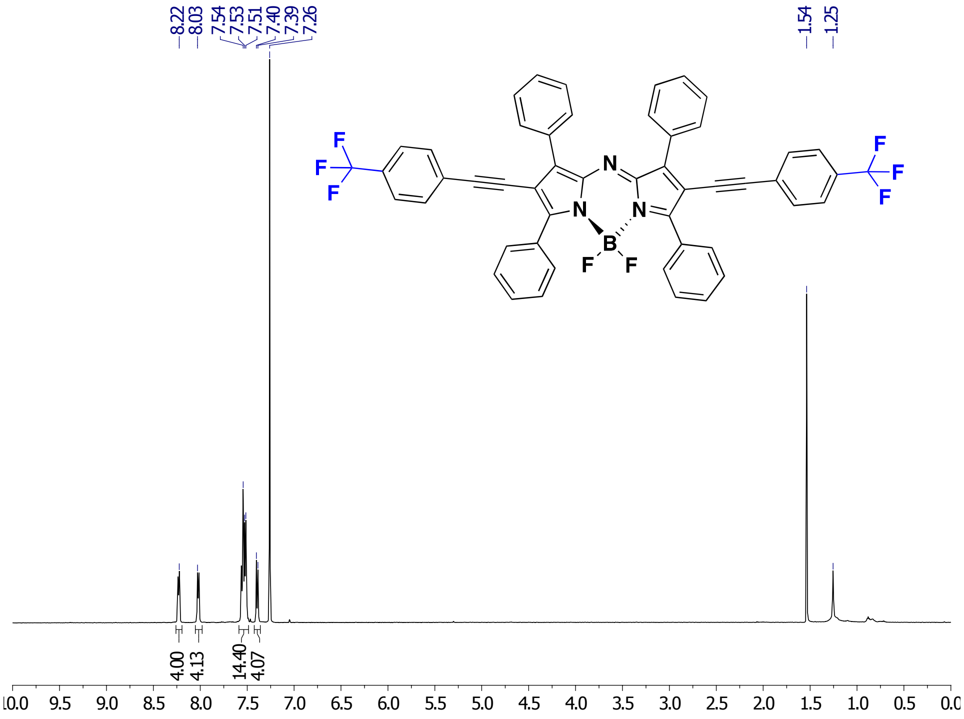

Figure S21 ^{1}H NMR of BF_2(L4)

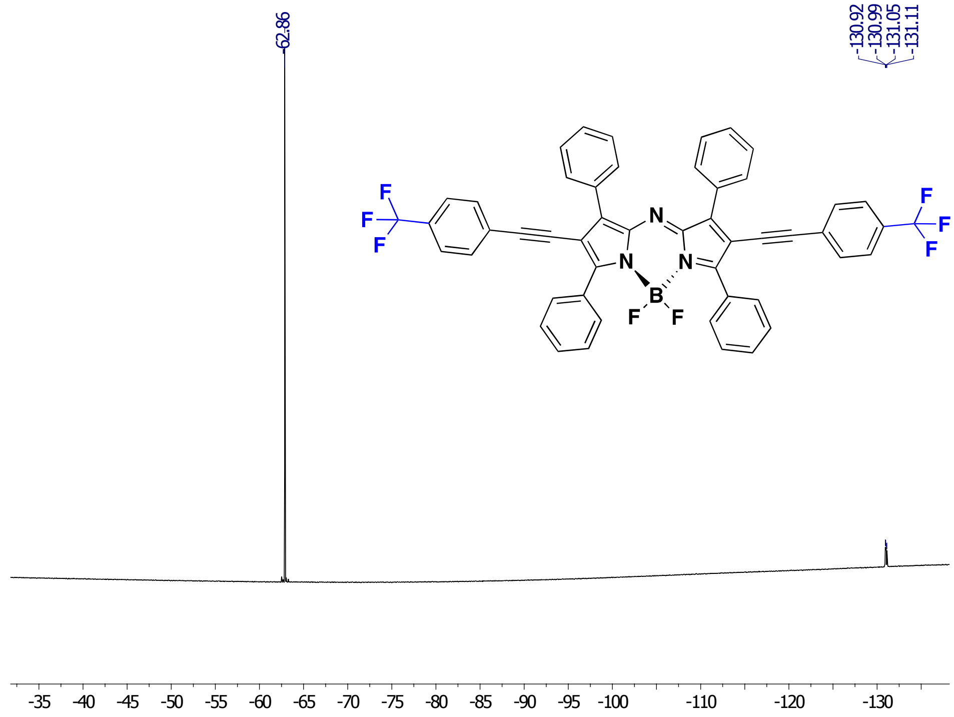

Figure S22 ^{19}F NMR of BF_2(L4)

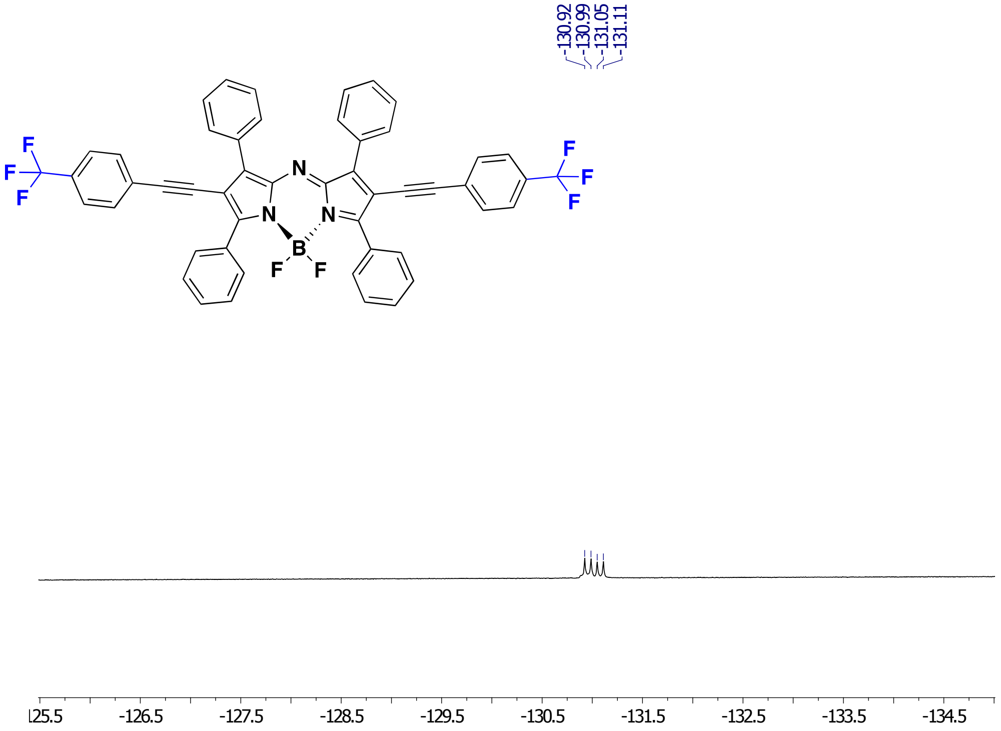

Figure S23 ^{19}F NMR of BF_2(L4), BF_2 peaks

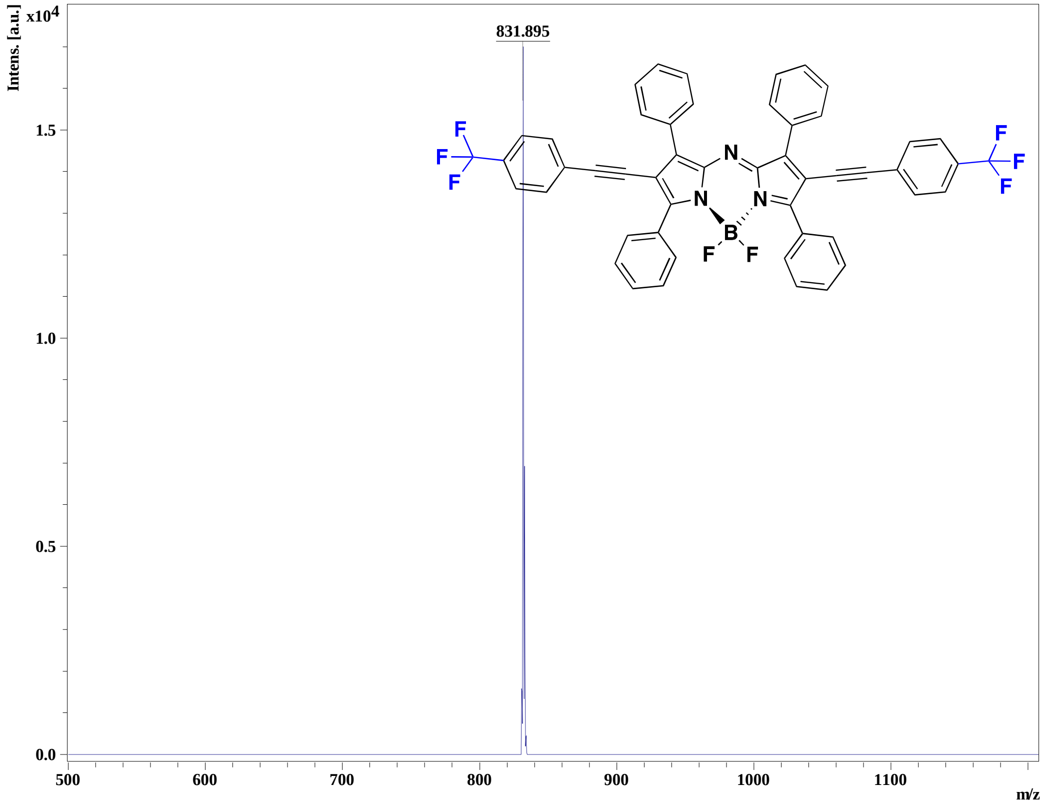

Figure S24 MALDI-TOF MS of BF_2(L4)

Zn(L1)$_2$

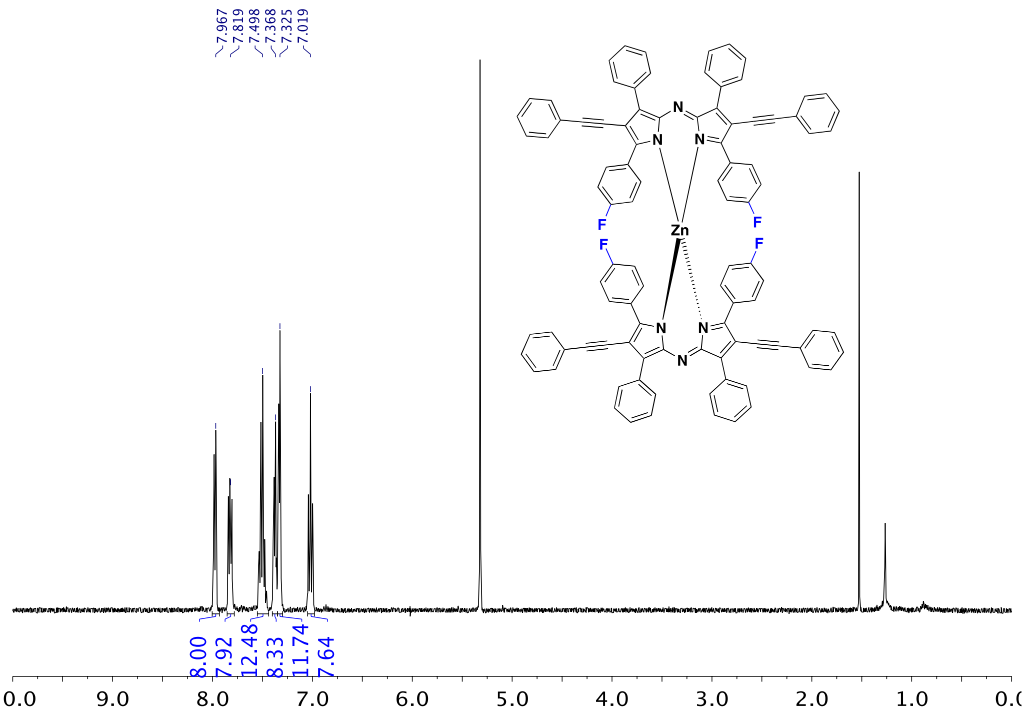

Figure S25 ^{1}H NMR of Zn(L1)$_2$

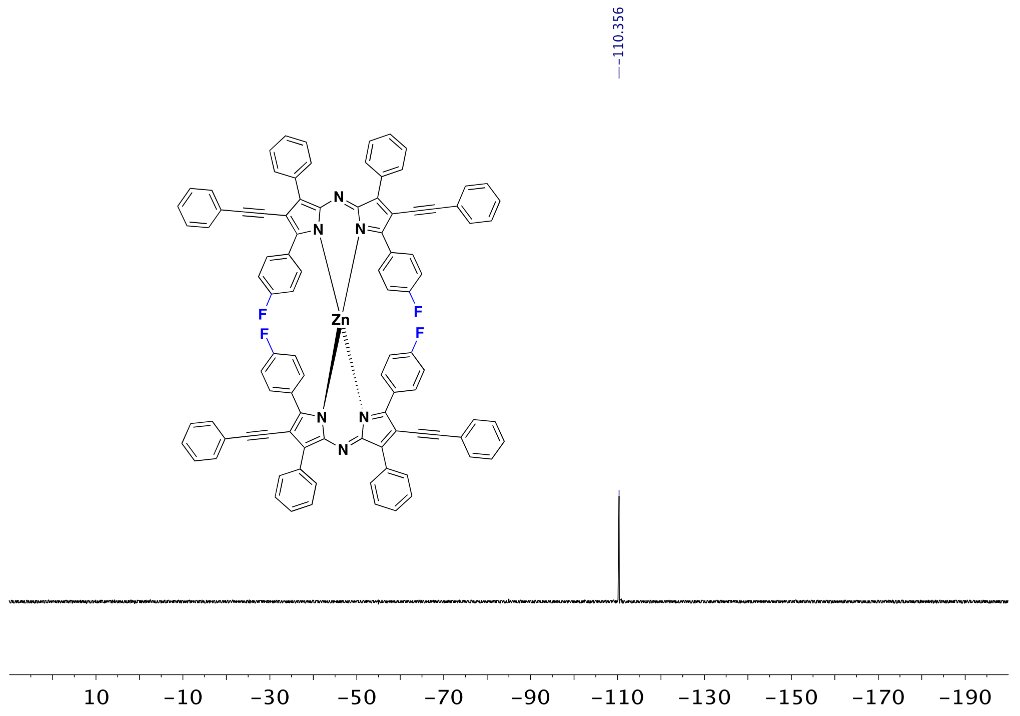

Figure S26 ^{19}F NMR of Zn(L1)$_2$

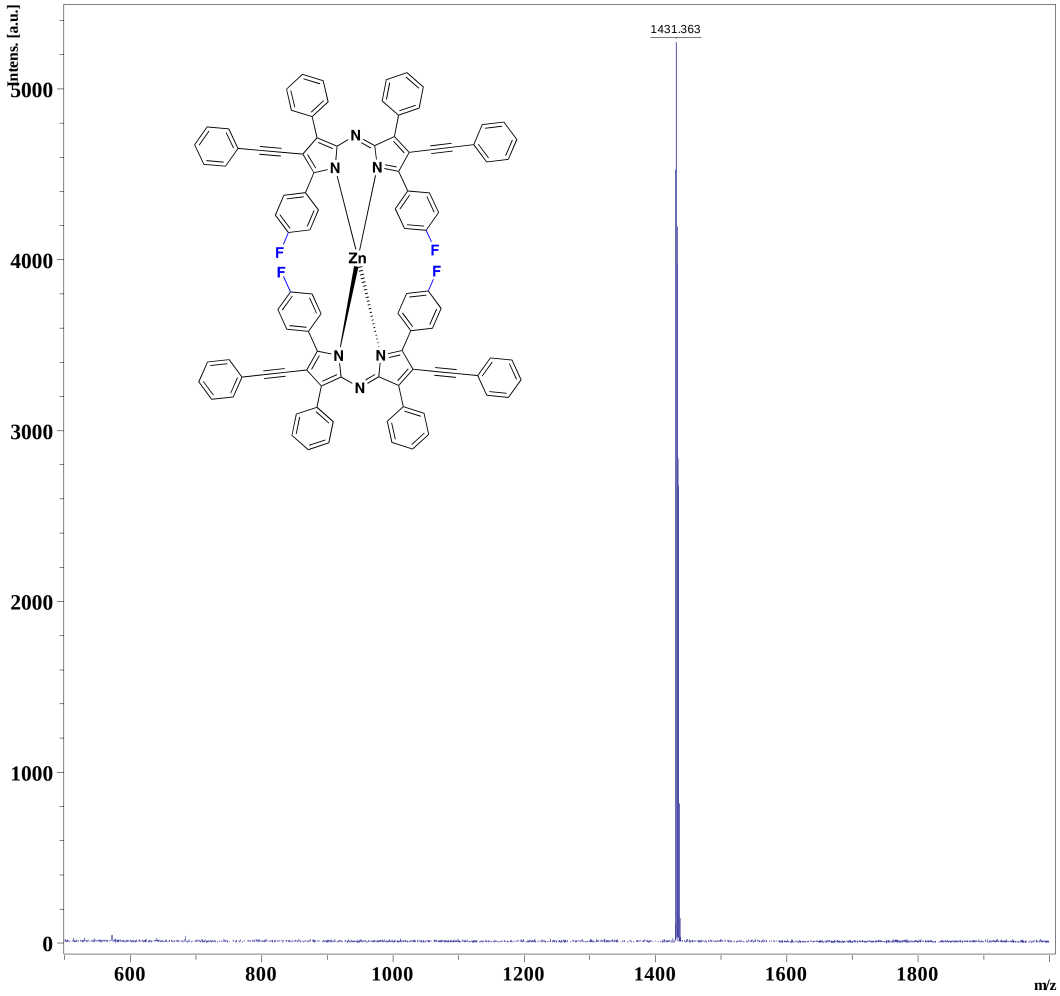

Figure S27 MALDI-TOF MS of $Zn(L1)_2$

Zn(L2)$_2$

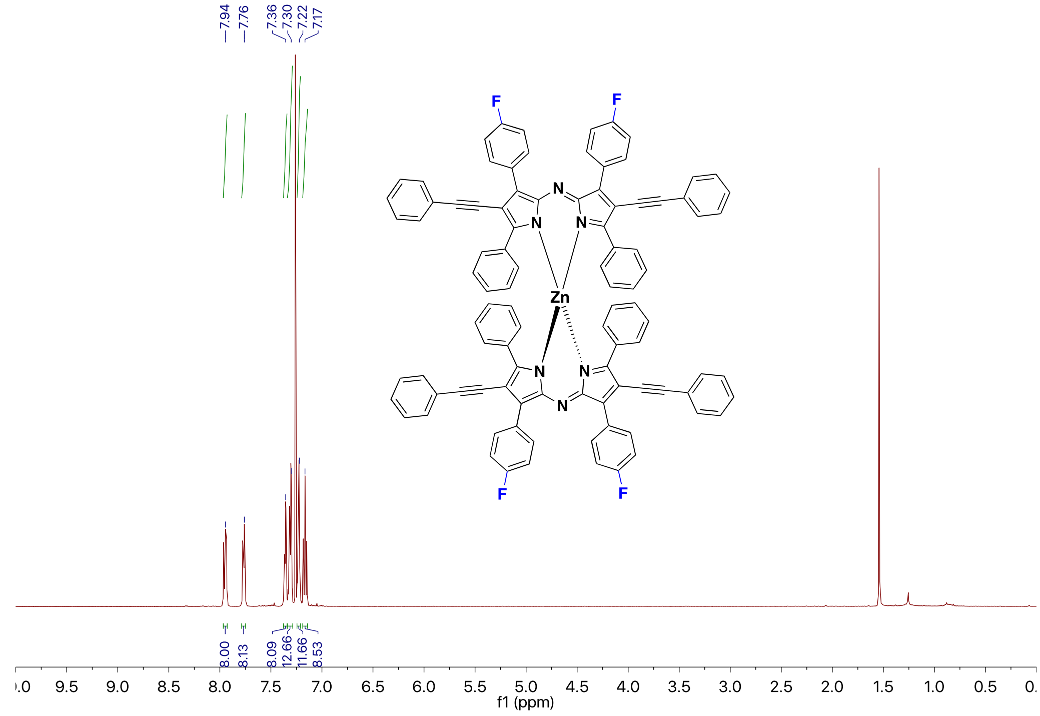

Figure S28 ^{1}H NMR of Zn(L2)$_2$

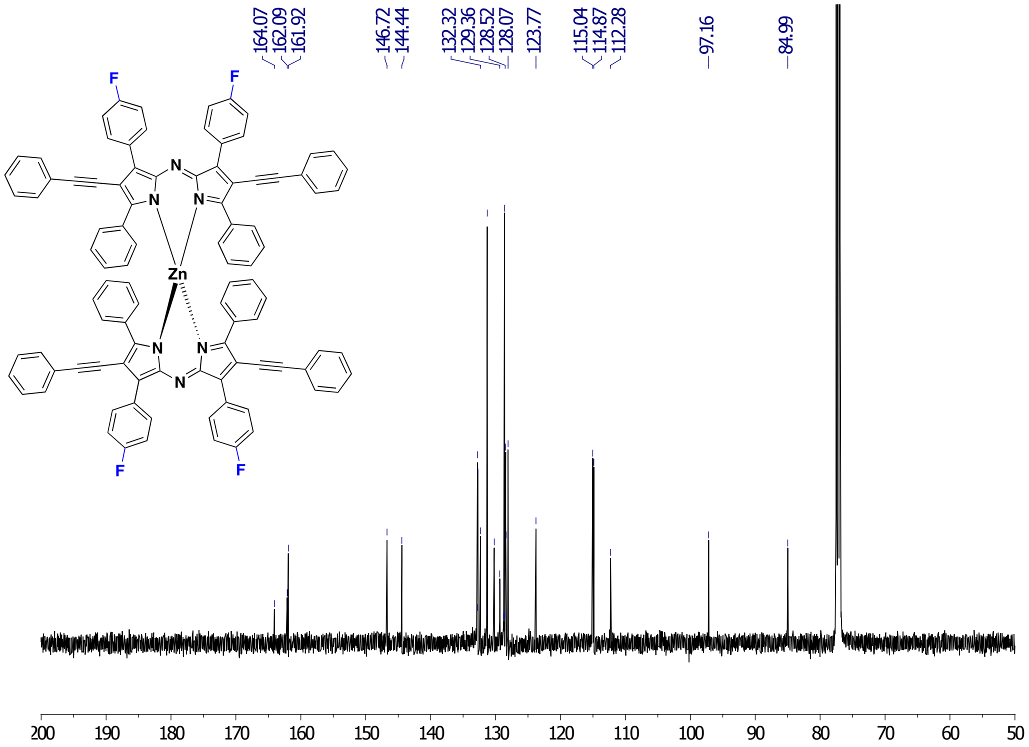

Figure S29 ^{13}C NMR of Zn(L2)$_2$

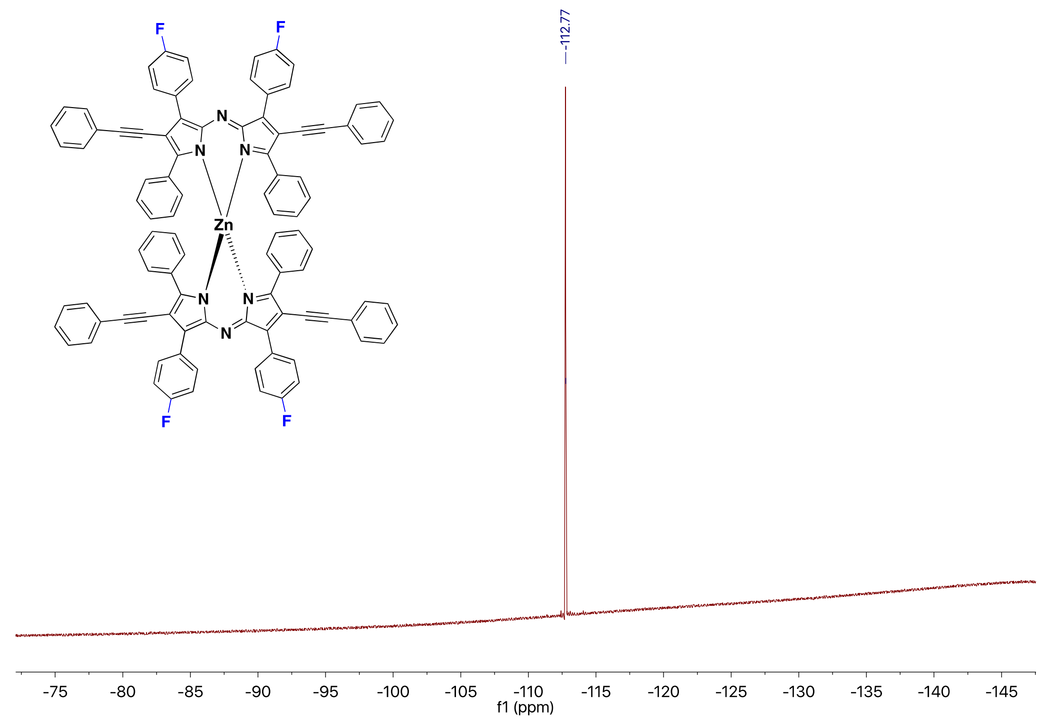

Figure S30 ^{19}F NMR of $Zn(L2)_2$

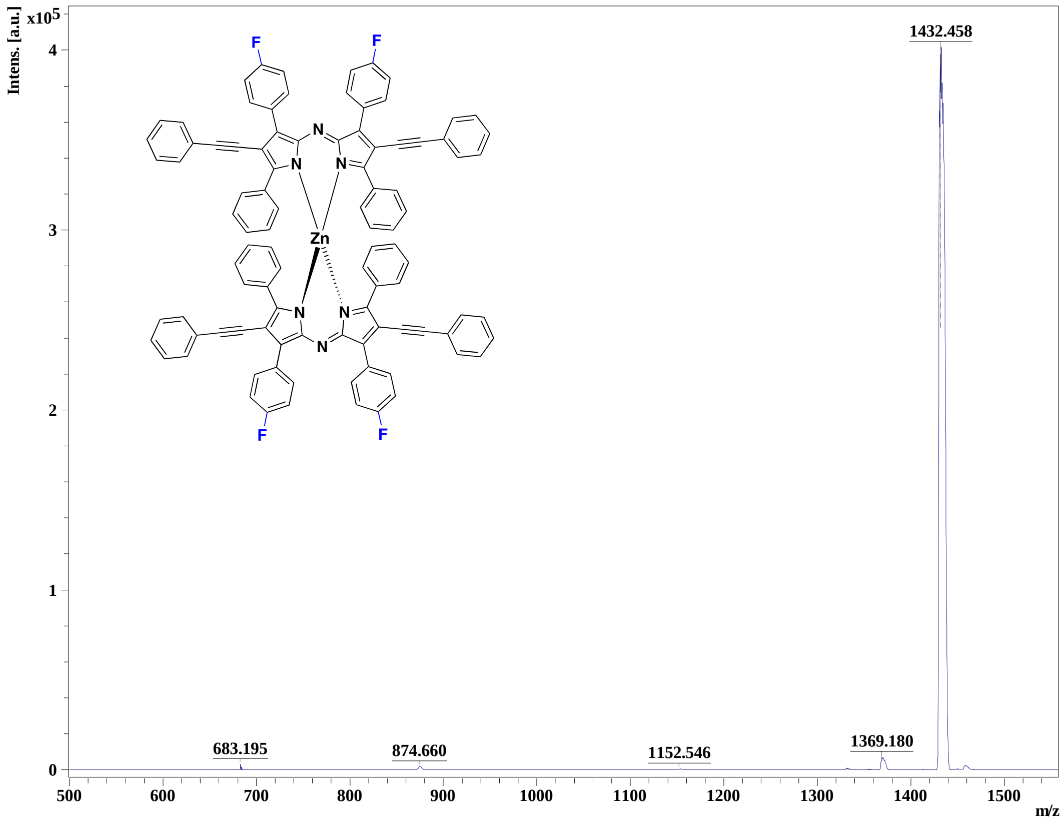

Figure S31 MALDI-TOF MS of $Zn(L2)_2$

Zn(L3)₂

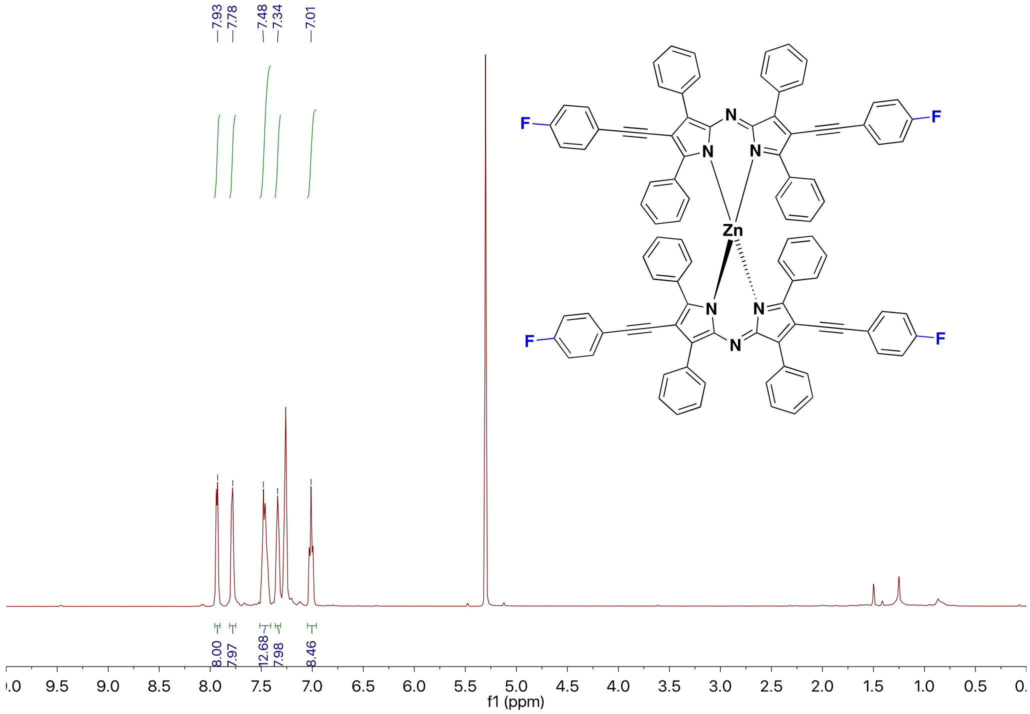

Figure S32 ^{1}H NMR of $Zn(L3)_2$

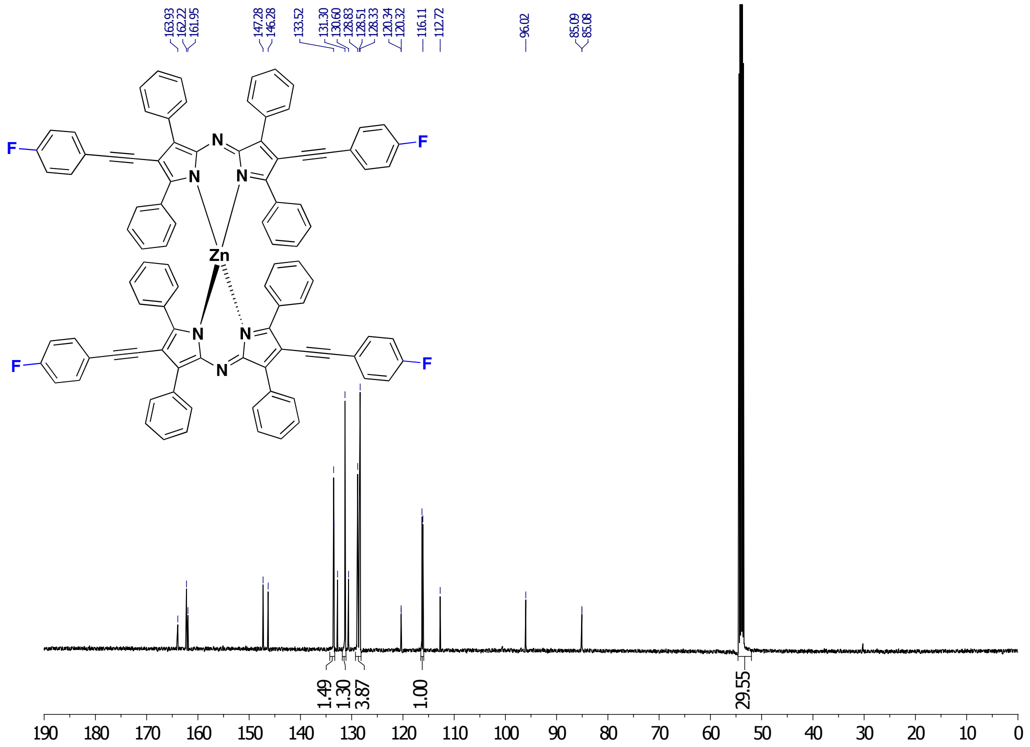

Figure S33 ^{13}C NMR of $Zn(L3)_2$

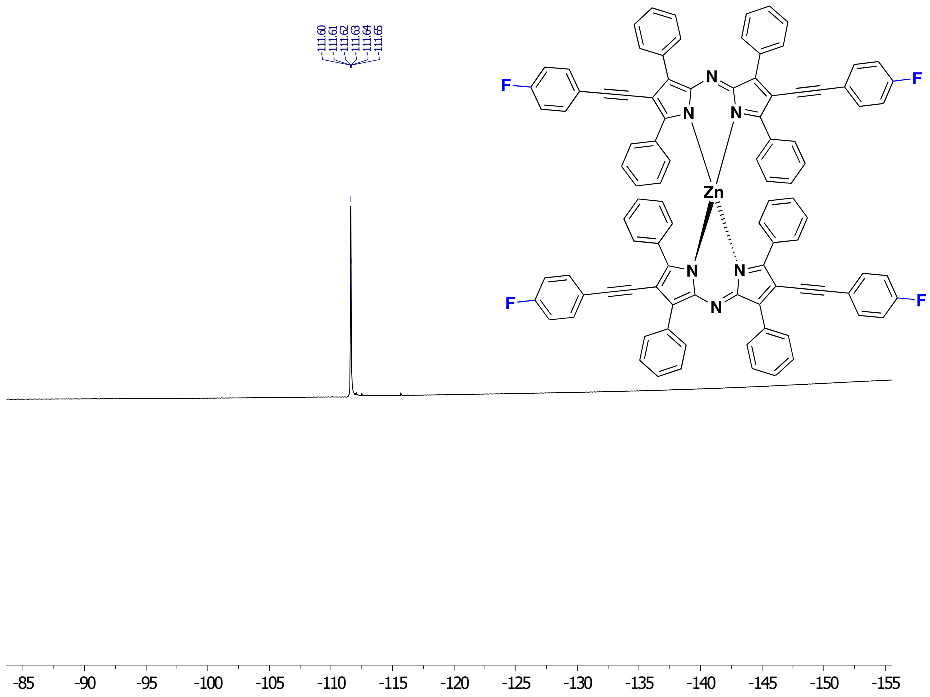

Figure S34 ^{19}F NMR of $Zn(L3)_2$

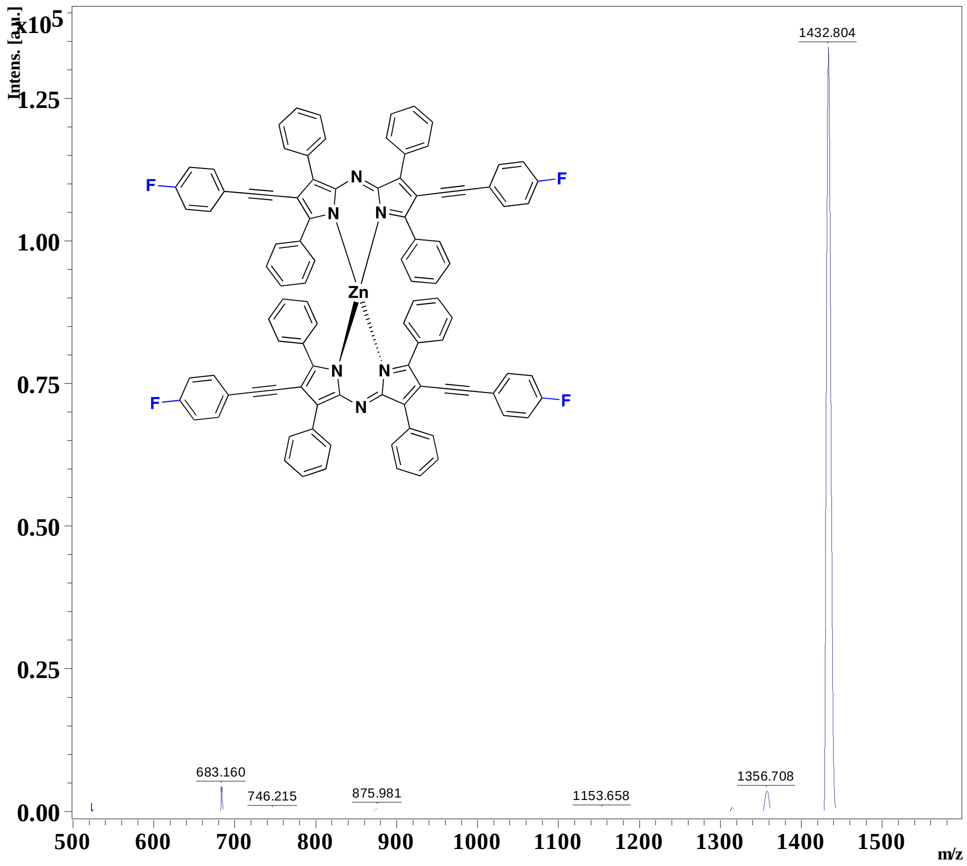

Figure S35 MALDI-TOF MS of $Zn(L3)_2$

Zn(L4)$_2$

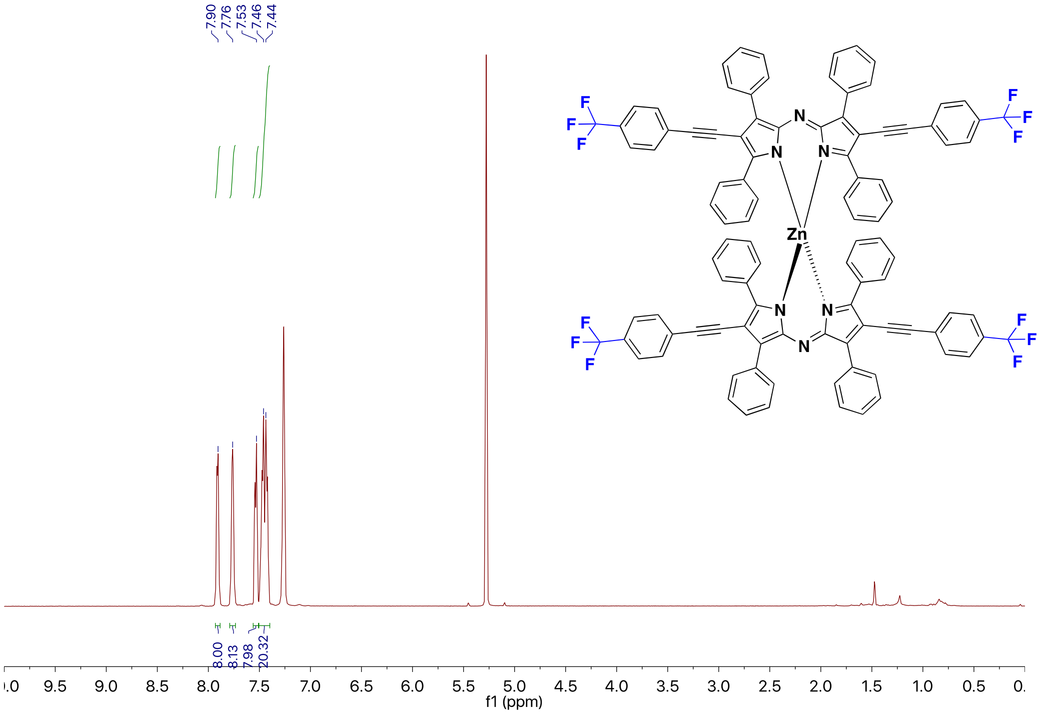

Figure S36 ^{1}H NMR of Zn(L4)$_2$

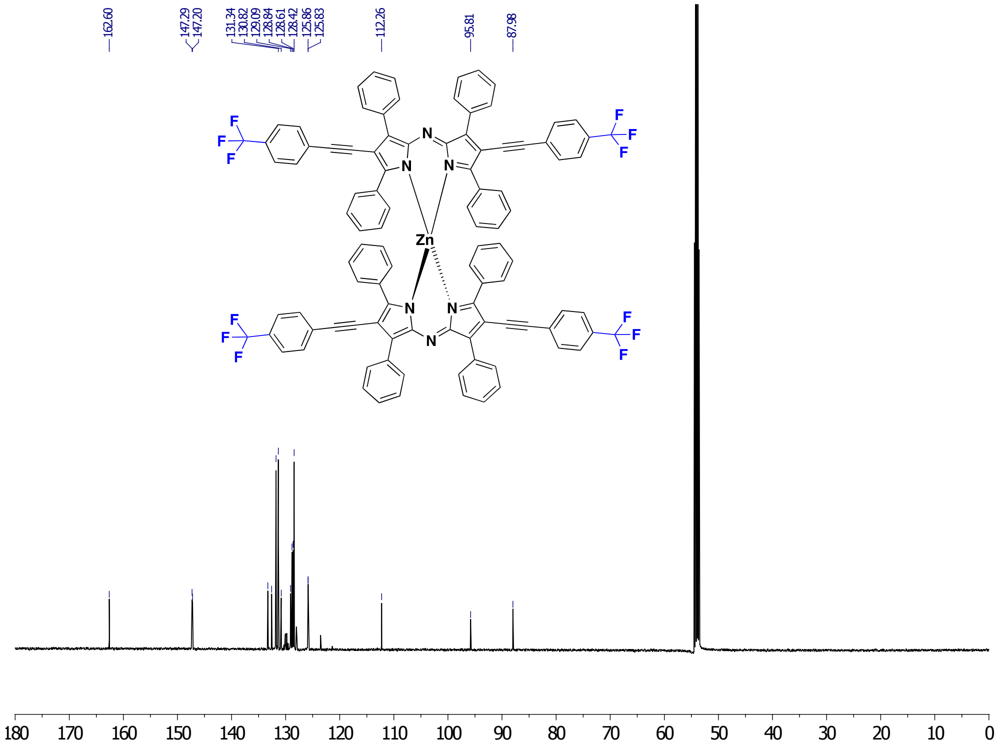

Figure S37 ^{13}C NMR of Zn(L4)$_2$

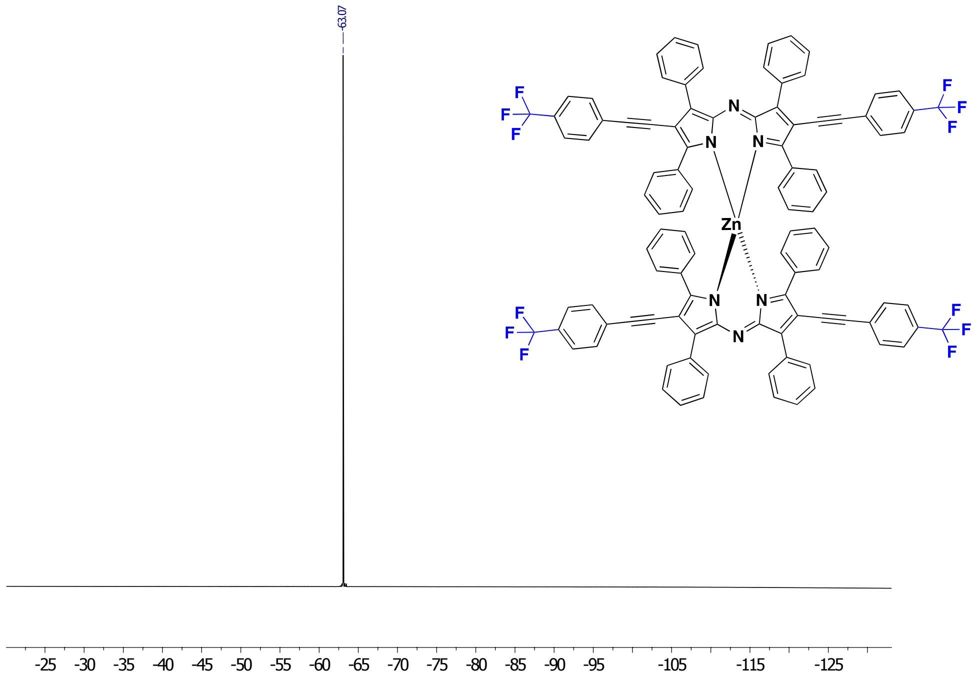

Figure S38 ^{19}F NMR of $Zn(L4)_2$

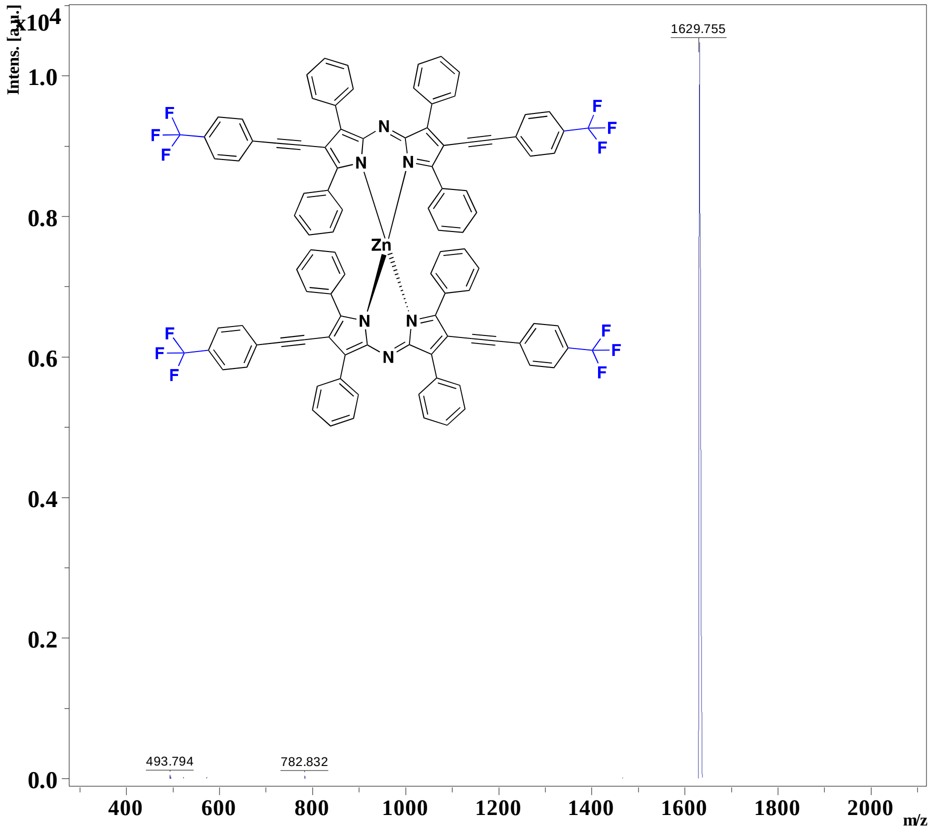

Figure S39 MALDI-TOF MS of $Zn(L4)_2$
